# Sex hormones and diets rich in polyunsaturated ω-6/ω-3 fatty acids modify microbiota distinctly in a mouse model of Alzheimer’s disease

**DOI:** 10.1017/gmb.2025.10005

**Published:** 2025-06-18

**Authors:** Lara Ordoñez-Gutierrez, Francisco Wandosell

**Affiliations:** 1 Departamento de Bioquímica y Biología Molecular, Universidad Complutense de Madrid, Madrid Spain; 2 Molecular Neuropathology Dept, Centro de Investigación Biomédica en Red de Enfermedades Neurodegenerativas (CIBERNED), Madrid, Spain; 3 Centro de Biología Molecular “Severo Ochoa” (CSIC-UAM), Universidad Autónoma de Madrid, Madrid, Spain

**Keywords:** Omega 3/6 Diets, Sexual Hormones, Amyloidosis, Aging

## Abstract

There is considerable data suggesting that the gut microbiota (GM) contributes to health and regulates host immunity and influences brain function, findings with implications for neurodegenerative diseases, such as Alzheimer’s Disease (AD).

In the present study, using three non-fat diets with different ratios of unsaturated ω-6/ω-3 fatty acids (FAs)(high or low), we analyzed how minor differences in diet can affect the microbiota of amyloid precursor protein/Presenilin 1 transgenic (APP/PS1 [TG]) mice, a mice model of AD, next, we studied how the levels of sex hormones may affect the GM. The data obtained show that sex hormones in males fed our standard diet (S) modified alpha and beta diversity, whereas no differences were observed in TG mice compared with wild-type mice. Moreover, there were significant differences in both alpha or beta diversity in mice fed with an H or L diet compared with an S diet.

In conclusion, our data indicate that the levels of sex hormones or differences in the ω-6/ω-3 FA ratio alter the GM more than expected. Thus, it is tantalizing to propose that low levels of ω-3 FAs in APP/PS1 mice fed an “H” diet may be responsible for modifying some bacterial genera, exacerbating the basal neuropathology in this AD model.

## Introduction

Recently, the GM has been shown to play a critical role in human health, in addition to its involvement in the degradation of macronutrients and the production of metabolites (Hooper et al., 2012). Numerous studies indicate that microbial communities are essential factors in many physiological processes including nutrition, inflammation, and protection against pathogens (Ivanov and Honda, 2012; Belkaid and Hand, 2014; O’Toole and Jeffery, 2015) The GM also produces some key compounds that modulate the innate immune system, affect intestinal epithelial cells, and alter endothelial cell function (Silva et al., 2020), generating a barrier that prevents the passage of antigens and bacteria from the gut into the bloodstream (Spadoni et al. 2015; Zhou et al., 2022). A growing body of clinical and experimental evidence suggests that the composition of the GM may also influence aging and influence brain disorders, such as Alzheimer’s disease (AD) (Cattaneo et al., 2017; Verhaar et al., 2022), a characteristic of which is the accumulation of amyloid plaques containing the amyloid-β (Aβ) peptide and fibrillary tangles composed of hyperphosphorylated tau. According to the amyloidogenic theory, the accumulation of Aβ-polymers induces oxidative stress, mitochondrial dysfunction, and synaptic impairment, provoking a glial response and neurodegeneration, and ultimately impairing cognitive performance (Hardy and Selkoe, 2002; Selkoe, 2002 Huang et al., 2016). A small percentage of AD cases have a genetic origin, familial AD (FAD) that is caused by mutations in the amyloid precursor protein (APP) (Mullan et al., 1992), Presenilin 1 and 2genes (Levy-Lahad et al., 1995) (Sherrington, 1996). However, more than 98% of AD cases are sporadic (of unknown cause). A genome-wide association study (GWAS) analysis of different sporadic cohorts highlighted more than 65 risk factors, including several cellular and metabolic elements that may be involved in AD (Bellenguez et al., 2022). Indeed, aging, environmental and/or dietary factors may have a strong influence on the appearance of this disease, and increasing evidence suggests an association between AD and certain metabolic disorders, including diabetes or hypercholesterolemia, which are also considered risk factors (Ricciarelli et al., 2012; Park et al., 2013; Ettcheto et al., 2015).

There is some clinical and preclinical evidence supporting the involvement of the GM in promoting AD onset and progression. For example, amyloid deposition has been associated with the presence of pro-inflammatory bacterial species in the gut and pro-inflammatory cytokines in the blood (Vogt et al., 2017; Zhuang et al., 2018). Preclinical studies showed that elements of the GM are necessary for brain amyloid deposition (Liu et al., 2019) and that the GM composition may be associated with neurodegeneration (Sheng et al., 2022; Verhaar et al., 2022). Significantly, antibiotic-mediated perturbations in the GM were proposed to modulate amyloid deposition in an AD mouse model (Minter et al., 2016), and an association between brain amyloidosis and pro-inflammatory gut bacteria was highlighted in cognitively impaired patients (Cattaneo et al., 2017; Fishbein et al., 2023; Munir et al., 2024). Some data suggests that the GM may influence brain physiology through signalling driven by the GM in this so-called microbiota–gut–brain axis (MGBA) (Frost et al., 2014; Vernocchi et al., 2016; Munir et al., 2024). Obviously, diet is an important element that can affect GM, and there is some evidence that specific diets may be correlated with AD (Morris et al., 2003; Scarmeas et al., 2006). Particular attention has focused on two major classes of polyunsaturated fatty acids (PUFAs), the omega-3 and omega-6 (ω-3/6) FAs (Burlingame et al., 2009; Sambra et al., 2021). Indeed, there are reports of a clear decrease in the risk of suffering AD and other dementias in people who consume large quantities of fish containing high levels of ω-3/6 PUFAs (Morris et al., 2003; Crawford and Broadhurst, 2012). The direct relationship between ω-3 FAs in the diet, mostly Docosahexaenoic acid (DHA-22:6 ω-3), and the evolution of a neuro-degenerative pathology has been addressed in animal models (Hooijmans et al., 2007; Cederholm and Palmblad, 2010; Herrera et al., 2019). Consequently, lower ω-3 FA levels in the diet have been linked to an increased risk of developing neurodegenerative diseases in rodent models and in humans (Ikemoto et al., 2001; Catalan et al., 2002; Barron et al., 2013). However, some clinical trials using ω-3 FAs in humans failed to establish a clear link (Barberger-Gateau, 2002). Another important consideration is the effect of gender reported. Even though a diet supplemented with DHA may reduce the hallmarks of AD in mouse models (Calon et al., 2004; Green et al., 2007; Perez et al., 2010), the presence of sex hormones may modify the final effect of DHA in some AD models at least. We previously found that high levels of DHA [22:6 ω-3] in the diet were not directly correlated with high levels of DHA in the brain cortex, both in male and female mice. However, the levels of sex hormones (oestrogen or testosterone) influence the effect of a DHA-rich diet on the amyloid burden (Herrera et al., 2018; Ordóñez-Gutiérrez et al., 2021). These data raise some questions about the effect of ω-6/ω-3-rich diets on the GM and on how ω-6 and ω-3 FAs modulate GM populations. Another question is how gender may influence the effect of a ω-6/ω-3 rich diet, particularly given that human aging is strongly associated with AD and with a physiological reduction in the levels of sex hormones.

As a result, a double-transgenic APPswe/PS1ΔE9 (Swedish mutant of Amyloid Precursor Protein, and deletion mutant of human presenilin 1), mouse AD model (TG) and their wild-type (WT) littermates were used here to determine how the GM is modified by changes in the levels of sex hormones, comparing male, castrated male, and female mice. In addition, the influence of diet on these alterations to the GM was assessed in animals fed a standard (**S**) diet or a low (**L)** or high (**H**) ω-6/ω-3 ratio diet. The data obtained show that the sex hormones do not appear to modify alpha and beta diversity in males following an **S** diet. However, when mice followed an **H** or **L** diet, there were significant differences in alpha and beta diversity relative to those following an **S** diet. This comparative analysis makes it tantalizing to propose that following an **H** diet enhances amyloidosis in males.

## Materials and methods

### Animals and husbandry

APP/PS1 TG mice were purchased from Jackson Laboratories (Bar Harbor; stock no. 005864), the B6.Cg-Tg [APPSwe, PSEN1dE9] (Swedish mutant of Amyloid Precursor Protein, and deletion mutant of human presenilin 1); 85Dbo/J (Mice strain C57BL/6 x C3H, transgeneinsertion 85), strain overexpressing the human APP gene carrying the Swedish mutation and exon-9-deleted presenilin 1 (PSEN1) (Jankowsky et al., 2001). The mice were housed at constant temperature (22 °C± 2 °C) and humidity (50% ± 5%) and on a 12:12 h light–dark cycle in a specific-pathogen-free animal facility. The orchiectomy procedure was performed following standard protocols (Sophocleous and Idris, 2019). All animal care and handling complied strictly with current Spanish legislation and guidelines and those of the European Commission [directive 2010/63/EU]. All the procedures involving the use and management of the TG mouse colony were approved by the Spanish Research Council (CEEA-CBMSO-33/307), the Community of Madrid (PROEX 341/15, recently extended by 5 years and Ref. PROEX 069.7/21), and the Spanish Research Council [CEEA-CBMSO-23/307.1].

### Genotyping

The genotype of the pups was confirmed by polymerase chain reaction (PCR) analysis (Ordóñez-Gutiérrez et al., 2015) using three primers: one anti-sense primer matching the sequence in prion (PrP) (5′ GTG GAT ACC CCC TCC CCC AGC CTA GAC C); one transgene specific sense primer (PS1 5’ CAG GTG GAG CAA GAT G; APP 5’ CCG AGA TCT CTG AAG TGA AGA TGG ATG), and a second sense-specific primer for genomic PrP (5’ CCT CTT TGT GAC TAT GTG GAC TGA TGT CGG). Only one PrP gene band, used as internal control, was detected in the WT mice, whereas three bands (APP, PS1, and PrP) were observed in the TG mice.

### Diets

Two diets were prepared by Up-Science [France] and in addition, our standard diet (**S**) was analyzed by the same company to determine the final percentage of each lipid (Supplementary Table 1A). Both the TG and WT genotypes used in these experiments (males, castrated males, and females) were fed these diets for 90 days, from 3 to 6 months of age. In all cases, the mice were allowed free access to food and tap water *ad libitum.* The experiments began from 3 months post-partum and the animal’s body weight was measured weekly. The **S** diet was gradually replaced by the corresponding experimental diet at the outset of the experiments (see below), the latter maintained until the animals reached 6 months of age (endpoint) when they were then sacrificed by CO_2_ inhalation.

- Standard food (**S**), as used in our animal facility, was purchased from Scientific Animal Food and Engineering (France), and it had a ω-6/ω-3 ratio of 12:3.
- The H diet (**H**), with a higher ω-6/ω-3 ratio, is an example of a very low ω-3 PUFA content diet. This pellet diet contains 34.5% ω-6 PUFAs and 1.4% ω-3 PUFAs, with a resulting ω-6/ω-3 ratio of 24:6.
- The L diet (**L**), had a lower ω-6/ω-3 ratio, in this case with fish-derived ω-3 PUFA, and with 30.2% ω-6 and 9.4% ω-3 PUFAs resulting in a ω-6/ω-3 ratio of 3:2.

None of these diets can be considered a “high-fat diet” as the total lipid content was similar to that of the **S** diet fat content. Total fat content: **L** 69 g/kg; **S** 46 g/kg; and **H** 73 g/kg (Ordóñez-Gutiérrez et al., 2021).

### Metagenomics

The metagenomics analysis was performed at the CBMSO Genomics and NGS Core Facility (GENGS) services, and the data were analysed with assistance from the Centro de Computación Científica – Universidad Autónoma de Madrid (CCC-UAM, https://www.ccc.uam.es/). The metagenomics experiment was set out to define the GM in 14 different experimental groups of mice, contemplating different sex hormone statuses, genotypes (WT or TG mice); and standard and high/low DHA content diets (**S, H,** and **L,** respectively). The experimental groups are as follows:

**Table 1:** 

General characteristics of the experimental groups				
Group^*^	Sex	Genotype	Diet	# Animals
WH	Male	WT	High DHA	5
WL	Male	WT	Low DHA	6
WS	Male	WT	Standard	6
TH	Male	TG	High DHA	3
TL	Male	TG	Low DHA	6
TS	Male	TG	Standard	6
WHC	Male castrated	WT	High DHA	6
WLC	Male castrated	WT	Low DHA	6
WSC	Male castrated	WT	Standard	5
THC	Male castrated	TG	High DHA	6
TLC	Male castrated	TG	Low DHA	6
TSC	Male castrated	TG	Standard	7
FW	Female	WT	Standard	5
FT	Female	TG	Standard	4

* The abbrevation represent the the combination of (Genotype-Diet). Sex differences were addedas “none indication” for male groups; C for castrated males and F for females.

### DNA isolation and 16S gene sequencing

DNA was extracted using the *DNeasy PowerLyzer* DNA isolation kit (Qiagen, Hilden, Germany) according to the manufacturer’s instructions. Bacterial gut biodiversity in these mice was assessed by amplifying the 16S region. The “complete sequence” includes the tails needed for Illumina indexing and sequence inclusion, and the “amplified sequence” includes the nucleotides to be trimmed from reads. The characteristics of the primers used are shown in (Supplementary Table 1B).

The data was analyzed using the MicrobiomeAnalyst 2.0 software; specifically, Qiime2 software5 was used for the metagenomics analysis, which is an open-source bioinformatics package to analyze the microbiome from raw DNA sequencing data (https://cutadapt.readthedocs.io/en/stable/). We used the q2-dada2 plugin. DADA2 is a complete pipeline to detect and correct Illumina amplicon sequence data, joining the reads, and to filter chimeric sequences (https://github.com/neufeld/pandaseq).

### Data availability

The sequencing data presented in this study can be found in the European Nucleotide Archive (ENA) online repository, accession number (# Study PRJEB79953) [https://www.ebi.ac.uk/ena/].

The data were organized into two sets:

- Subset 1 (**S Diet**): mice on a standard diet. This subset will allow the differences in the GM to be defined depending on sex/circulating hormones and genotype under normal metabolic status, as the diet is unmodified.
- Subset 2 (**Males**): males only. This subset will allow the effect of altering diet on the GM to be determined, as well as the effect of the presence/absence of gonads and the genotype in one gender.

To compare the GM composition between the groups, we first used α-diversity and β-diversity parameters, using the rarefied microbiota data to calculate the alpha and beta diversity. As a general rule, we used “phylum level” and “genus level” as the standard analysis, and only in a few cases “feature level” was represented.

A qualitative and statistical analysis of subsets 1 and 2 were performed with the MicrobiomeAnalyst 2.0 web software (https://www.microbiomeanalyst.ca/). The α-diversity parameter is evaluated at the feature (operational taxonomic unit [OTU]/amplicon sequence variant [ASV]) level using Shannon, and significant differences are evaluated using Mann–Whitney/Kruskal–Wallis, (posthoc) pairwise comparisons, test for continuous variables with non-normal distributions.

Beta-diversity: For beta-diversity analysis, we used principal coordinate analysis (PCoA) plot. By default, the difference in diversity is assessed using the Bray–Curtis index. When comparing two (or more) groups, we used a permutational multivariate statistical analysis of variance (PERMANOVA) (Pairwise PERMANOVA). We used this route: BIOM format and QIIME v1.5.0. Data filtering: A 20% prevalence filter means at least 20% of its values should contain at least 4 counts; and a low variance filter of 10% was applied, using inter-quantile range (IQR). The normalization consists of the following options: (1) Data rarefying was performed by randomly subsampling without replacement to the size of the smallest library that is not considered defective”. (2) Data scaling: total sum scaling (TSS), cumulative sum scaling (CSS), upper-quantile normalization (UQ). 3) Data transformation: relative log expression (RLE), trimmed mean of M-values (TMM), centred log ratio (CLR). Rarefaction was performed with all samples. Performed total sum normalization. No data transformation was performed.

In addition, we used Microbiome Analyst software to establish principal components plot, the linear discriminant analysis (LDA, scores ≥2), and the linear discriminant analysis effect size (LEfSe), as well as a single-factor statistical comparisons (DESeq2) to identify the specific biomarker group. This analysis focusses on both the statistical significance and biological relevance, helping identify biomarkers that exhibit substantial differences between the groups. A Mann–Whitney test was used for single-factor statistical comparisons, and the Kruskal–Wallis test was used to assess whether 2 or more samples come from the same distribution. The abundance of the most significantly altered genus among the experimental groups was represented as log-transformed values, derived from the LEfSe or single-factor statistical comparisons data [false discovery rate (FDR) <0.05].

## Results

### Comparative analysis of the microbiota in WT and TG mice fed a standard (S) diet

**Figure 1.**
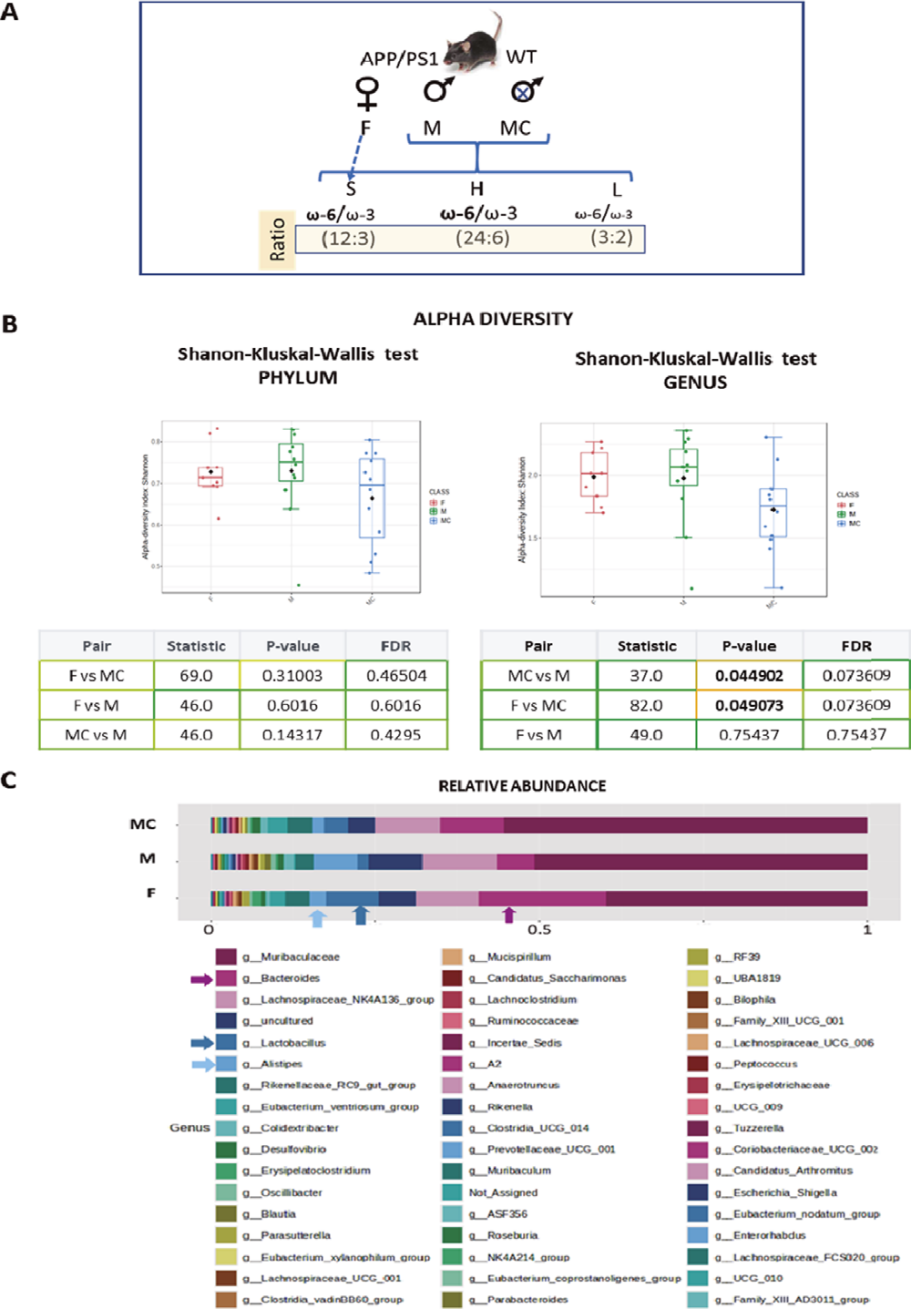
**(A)** Schematic representation of the experiments with different genotypes and diets. We fed APP/PS1 double transgenic mice and wild-type littermates; castrated (**MC**) and non-castrated (**M**) with three different diets (**S, H,** and **L**), containing different **ω-6/ ω-3** ratios. In addition, a group of APP/PS1 and WT females fed only with the **S** diet was used as control having the full repertoire of sexual hormones. **(B)** Plots show the alpha analysis from the three sexual groups considered (F, M, and MC), using Shannon test. The below data indicate the Kruskal–Wallis statistical analysis in which we represented *p* and DFR values. **(C)** The stacked bar plots represent the relative abundance profile in F, M, and MC mice groups, at genus taxonomic rank. Some obvious differences were marked with colour arrows.

The GM data from WT and TG mice was analyzed using the Shannon test to address variability in metagenomics samples, a test that considers richness and distribution [evenness], while the beta diversity was analysed with the Bray–Curtis dissimilarity test. Our first aim was to determine whether differences in the GM could be detected between TG and WT mice fed the standard diet used at our animal facility (**S**), analyzing samples obtained from male and female mice of both genotypes (See all schematic representations in Figure 1A). The alpha analysis showed a similar level of complexity between WT and TG mice, with no significant differences at the phylum or genus level (Supplementary Figure 1A). The relative abundance analysis showed that *Patescibacteria* were more abundant in WT samples (Supplementary Figure 1B), while at the genus level, *Alistipes*, or *Desulfovibrio* were more abundant in the TG samples (Supplementary Figure 1B).

When the beta diversity was assessed between both genotypes, no major differences were detected at the phylum level (Permanova test, *p* = 0.787) or at the genus level (Permanova test, *p* = 0.494; Supplementary Figure 1C).

Subsequently, the LEfSe was used to identify genomic features that differed significantly between each group in our metagenomic analyses (i.e., phylum, genus, and species). At the family and genus level, only g-RF39 was more strongly represented in TG samples relative to the WT male mice (*p* = 3.96E−4: Supplementary Figure 1D), as further confirmed using a single-factor statistical comparison (Mann–Whitney/Kruskal–Wallis test, *p* = 4.31E−4; Supplementary Figure 1E).

### Sex hormones and diversity

Within the group maintained on an **S** diet, we determined whether different levels of sex hormones (testosterone and derivatives) might affect the GM. Three groups of animals were established, females (**F**), males (**M**), and castrated males (**MC**) (Scheme Figure 1A), and when these groups were considered, the Shannon index failed to detect significant differences among the groups at the phylum level. By contrast, differences between **MC**, and **M** or **F** were evident at the genus level (*p* = 0.044902 and 0.049073 respectively, Kruskal-Wallis: Figure 1B). By analyzing the relative abundance in these groups, at the phylum level, *Proteobacteria* were more abundant in **F** than in **M** mice, and more than in **MC** mice (**F > M > MC**: Supplementary Figure 2A). At the genus level, we detected clear differences and for instance, g-*Bacteroides* and g-*Lactobacillus* were more abundant in **F** than in **MC** mice, and lower in **M** (**F > MC > M**). By contrast, some genera were more abundant in **M**, such as g-*Alistipes* or g-*Blautia*, and they were clearly reduced in **F** and **MC** mice (Figure 1B).

**Figure 2.**
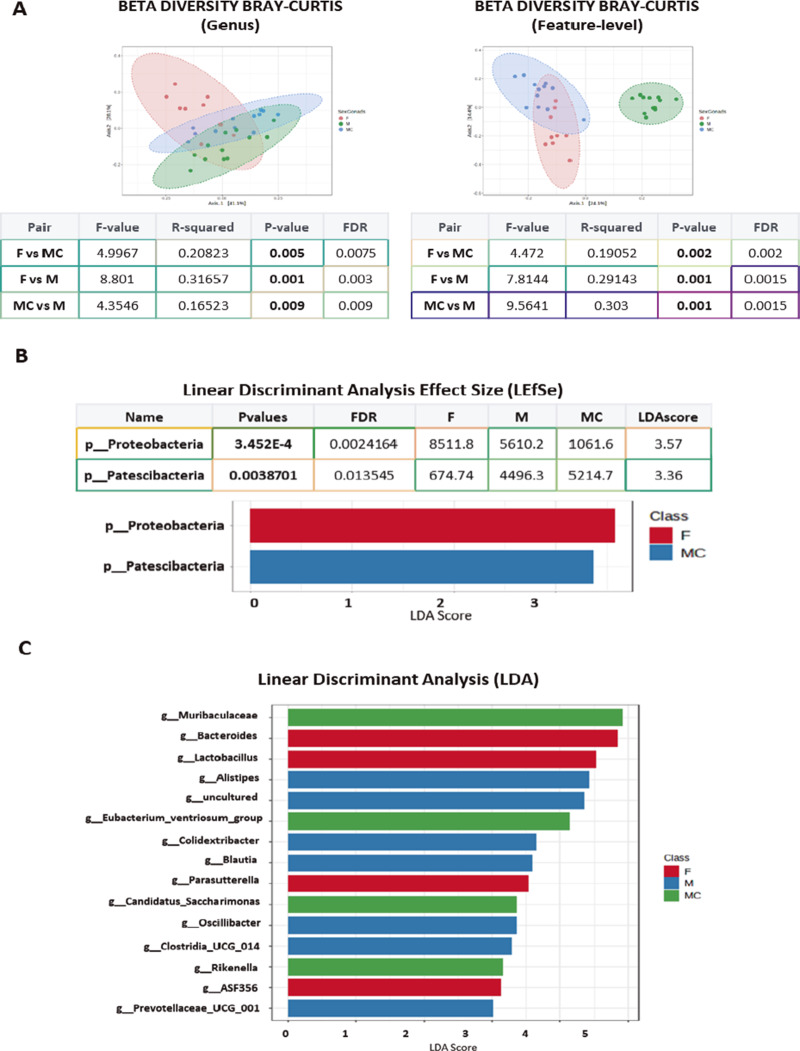
**(A)** Plots show beta diversity analysis from sexual mice groups considered (F, M, and MC), using Bray–Curtis, at genus and feature level. The corresponding statistical analysis is indicated below, representing *p* and DFR values. **(B)** The data from sexual mice groups considered (F, M, and MC) were additionally analyzed using linear discrimination test (LEfSe) showing two phyla significantly different, one in the F group and one in the MC group. **(C)** Plots show linear discrimination analysis (LDA) from F, M, and MC mice. Data show some genera represented when considering LDA >2.

The analysis of beta diversity failed to identify significant differences at the phylum level (Supplementary Figure 2B), whereas the three groups presented differences at the genus level (Permanova test – **F** vs **MC**, *p*-value 0.005, **F** vs **M**, *p*-value 0.001; **MC** vs **M**, *p*-value 0.009) and at the feature level (Permanova test – **F** vs **MC**, *p*-value 0.002; **F** vs **M,** *p*-value 0.001; **MC** vs **M**, *p*-value 0.001: Figure 2B).

Through a LEfSe analysis, we found a significant increase in *p*-*Proteobacteria* in **F** and *p*-*Patescibacteria* in **MC** at the phylum level (Figure 2B), whereas several differences were observed at the genus level associated with the gonads, such as those in g-*Blautia* in **M**, g-*Lactobacillus* in **F**, or g-*Candidatus Saccharimonas* in **MC** (Figure 2C and Supplementary Figure 2C). At the species level, the data confirmed the increase in the presence of *Bacteroides caecimuris* and *Lactobacillus reuteri* in **F** mice, which might represent a “female mouse signature” (Supplementary Figure 3).

### Comparative analysis of microbiota between WT and TG mice following a standard diet and the effect of sex hormones

#### The effect of different sex hormones

**Figure 3.**
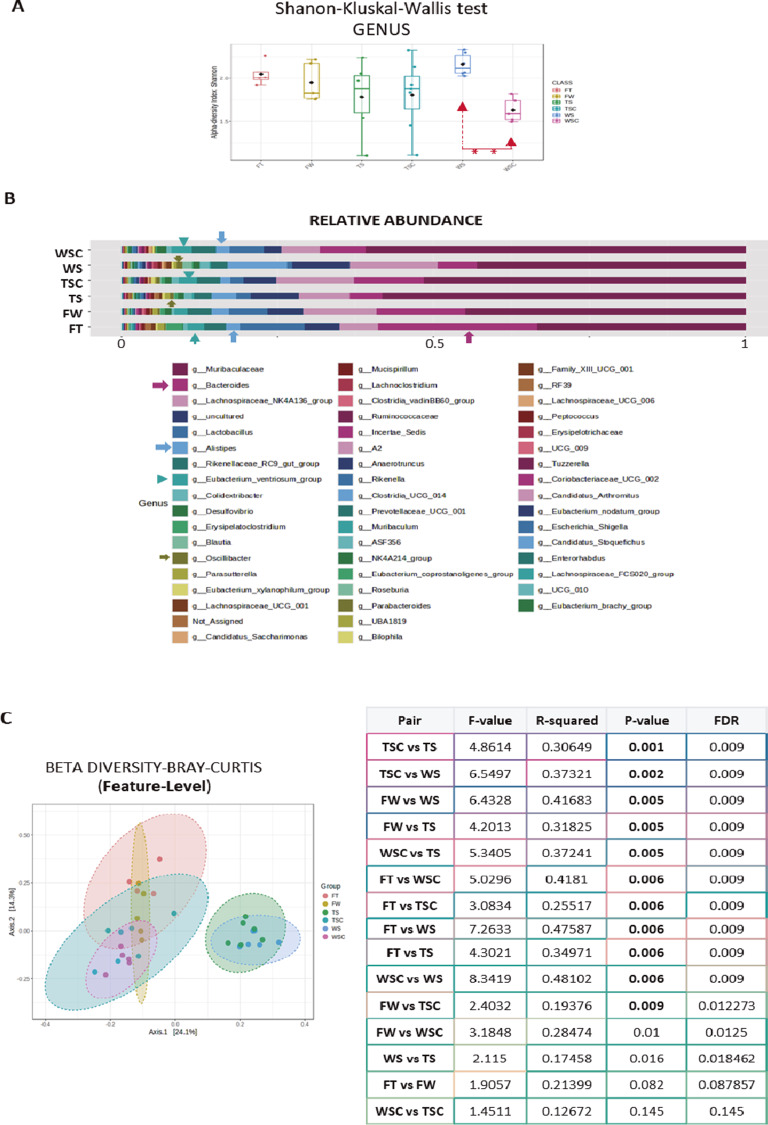
**(A)** Plots show the alpha analysis from the three sexual groups and the two genotypes considered (FW, FT, WS, WSC and TS, TSC), using Shannon test. The asterisks (**- *p* < 0.001) represent the *p* value of the Kruskal–Wallis statistical analysis. **(B)** The stacked bar plots represent the relative abundance profile in FW, FT, WS, WSC and TS, TSC mice groups, at genus taxonomic rank. Some obvious differences were marked with colour arrows. **(C)** Plots show beta diversity analysis from FW, FT, WS. WSC and TS, TSC mice groups, using Bray–Curtis, at feature level. The corresponding statistical analysis was represented on the right table, representing *p* and DFR values. (Note that *p* values were represented in bold only when with FDR below 0.03.)

To extend our data on the effect of diet on the GM, we set out to assess the influence of sex hormones by considering six experimental groups: WT females (**FW**) and TG females (**FT**), WT (**WS**) and TG (**TS**) male mice and, and WT (**WSC**) and TG (**TSC**) castrated male mice. An alpha analysis identified some significant differences at the genus and species levels, although we only considered the differences between **WS** and **WSC** to be particularly relevant (*p* = 0.00433, FDR = 0.064: Figure 3A, Supplementary Figure 4A). Histograms highlighted the alterations in GM composition, with a reduction in the phylum *Proteobacteria* in **WSC** and **TSC** mice (Supplementary Figure 4B). At the genus level, there were also some clear differences, for instance, there was a reduction in *Alistipes* in **TSC** and **WSC** mice when compared with **TS** relative or **WS** respectively. The genus *Bacteroides* was more abundant in female TG animals (**FT > FW**) and in castrated males (**TSC** and **WSC**), whereas *Oscillibacter* was more abundant in males (**TS** and **WS**) than in females (**FW** and **FT**: Figure 3B).

The beta diversity analysis showed no major differences at the phylum level but significant differences at the genus level, for instance, when comparing **WS** with either **WSC** (*p*-value = 0.006) or against **FW** and **FT** (*p*-value = 0.005 and 0.006, respectively**:** Supplementary Figure 5A). When a Bray–Curtis comparison was performed at the group feature level, almost all groups were significantly different except for **FT** vs **FW** or **WSC** vs **TSC** (Figure 3C).

An LDA at the genus level identified differences in g-*Blautia*, g-*Alistipes*, and g-*Oscillibacter* in **WS** mice or in g-*Rikenella*, g-*Candidatus Saccharimonas*, and g-UBA1819 in **WSC** mice, among others (Supplementary Figure 5B). A more extensive LEfSe analysis showed a significant increase in *p*-*Proteobacteria* in **FT** mice and *p*-*Patescibacteria* in **WSC** mice at the phylum level, in accordance with the previous data (Supplementary Figure 6). Moreover, at the genus level, 20 significantly different changes were identified (Supplementary Figure 6), including those to g-*Blautia*, g-*Alistipes*, g-*Lactobacilus.* Some specific analyses were performed using single-factor statistical comparisons (EdgeR), showing significant differences in genera between **WS** and **FW** mice (i.e., g-*Lactobacillu*s or g-*Clostridia* UCG014) or **WS** and **TS** mice (g-RF39 or g-*Eubacterium*_brachy_group) as well as an effect of testosterone depletion in **WS** vs **WSC** mice (i.e., g-*Lactobacillus* or g-*Peptococcus* or g-*Eubacterium ventriosum* group: Supplementary Figure 7).

### The effect of diets containing different amounts of unsaturated ω-3/6 fatty acids

The second aim of this work was to analyze the differential effects of three diets that had a similar total fat composition but with different levels of ω-6/ω-3 PUFAs. We previously reported that these diets modified the amyloid burden in APPSWE/PS1ΔE9 male and female mice. Hence, here, we studied the populations of mice considered above (males, castrated males, and female mice of both genotypes – **WT** and **TG**) when they were fed with these diets: standard diet (**S**), low (**L**), and high (**H**) ω-6/ω-3 ratio diets. We first performed an alpha analysis that took into account both genotypes, which highlighted some differences in GM abundance. Significant differences were evident at the phylum level (**WT** vs **TG**, *p* = 0.014), for instance with a clear reduction of *p*-*Patescibacteria* (Supplementary Figure 8A–B). Likewise, at the genus level, g-*Muribaculaceae* was more abundant in **TG** mice, whereas g-*Alistipes* and g-*Mucispirillum* were present in greater quantities in **WT** mice (Supplementary Figure 8C). Similarly, the alpha analysis of castrated versus non-castrated males showed statistical differences at both the phylum and genus levels (Supplementary Figure 9A). In terms of relative abundance, there was a clear reduction in *p*-*Patescibacteria* and *p*-*Deferribacterota* in **MC** mice, whereas, at the genus level, g-*Muribaculaceae* was more abundant in **MC** mice, whereas g-*Blautia*, g-*Alistipes*, g-*Rikenellaceae*-RC90 or g-*Mucispirillum* were more abundant in **M** mice (Supplementary Figure 9B–C).

In terms of beta diversity, no major differences were evident at the phylum or genus level between **WT** and **TG** mice (Supplementary Figure 10A), whereas there were significant differences at the feature level between **MC** and **M** mice (*p* = 0.004) and almost significant differences at the genus level (*p* = 0.057, FDR = 0.057: Supplementary Figure 10B). The subsequent LDA analysis showed the genera g-*Romboutsia* and g-*Rikenella* were more abundant in **MC** mice, also remaining as the most abundant genera in **M** mice (Supplementary Figure 10C). The additional LEfSe statistics showed that *p*-*Proteobacteria* and *p*-*Deferribacterota* were significantly more abundant in **M** mice at the phylum level, and 10 genera were significantly different, with g-*Rikenella* and g-*Romboutsia* most abundant in **MC** mice (Supplementary Figure 10D).

**Figure 4.**
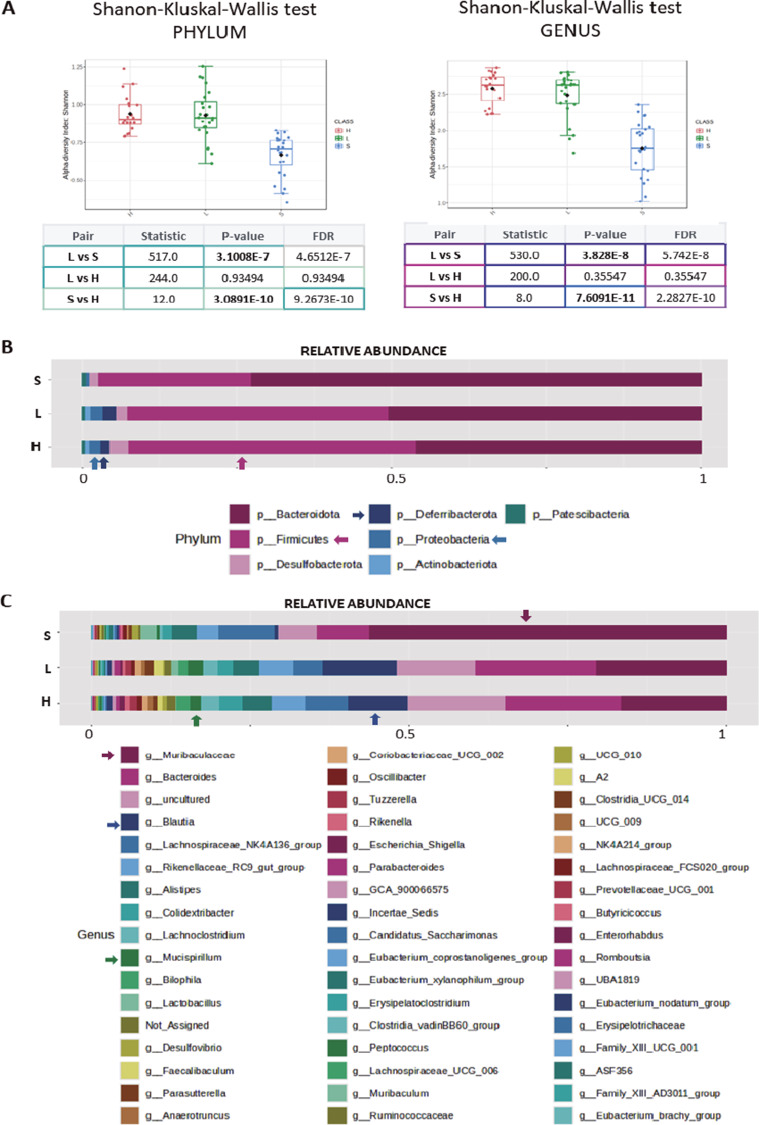
**(A)** Plots show the alpha analysis at phylum and genus level, from the three diets used (S, L, and H), using Shannon test. The table represents the *p* value and FDR value from the Kruskal–Wallis statistical analysis. (Note that *p* values were represented in bold only when with FDR below 0.03.) **(B)** The stacked bar plots represent the relative abundance profile of microbiota after feeding with S, or L, or H diets at phylum taxonomic rank. Some obvious differences were marked with colour arrows. **(C)** The stacked bar plots represent the relative abundance profile of microbiota after feeding with S, or L or H diets at genus taxonomic rank. The colour arrows match with the corresponding genera in the list, and some evident differences were obvious.

An alpha analysis was then performed that focused on the three diets, and the data showed, at the phylum level, that the **S** diet had a significant effect relative to both the **L** and **H** diet (S vs L – 3.1 E−7; S vs H – 3.089 E−10), with no significant differences between the **L** and **H** diet (Figure 4A, left panel).

At the genus level, the differences between the **S** and **L** or the **S** and **H** diets were very significant (S vs L – 3.82 E−8; S vs H – 7.609 E−11) in contrast to those between the **H** and **L** diet (Figure 4A, right panel). The analysis of relative abundance showed that *p*-*Bacteroidota* was more abundant in mice following the **S** diet, whereas *p*-*Firmicutes*, *p*-*Deferribacterota*, and *p*-*Proteobacteria* more abundant in the animals following an **H** or **L** diet (Figure 4A–B). Differences were evident at the genus level, for instance, in the greater abundance in g-*Blautia* and g-*Lactobacillus* in animals following a **H** or **L** diet. As a general rule, both the mice following an **H** and **L** diet had a wider distribution of some genera due to the relative reduction of g-*Muribaculaceae*, which was more abundant in those following an **S** diet (Figure 4B–C).

**Figure 5.**
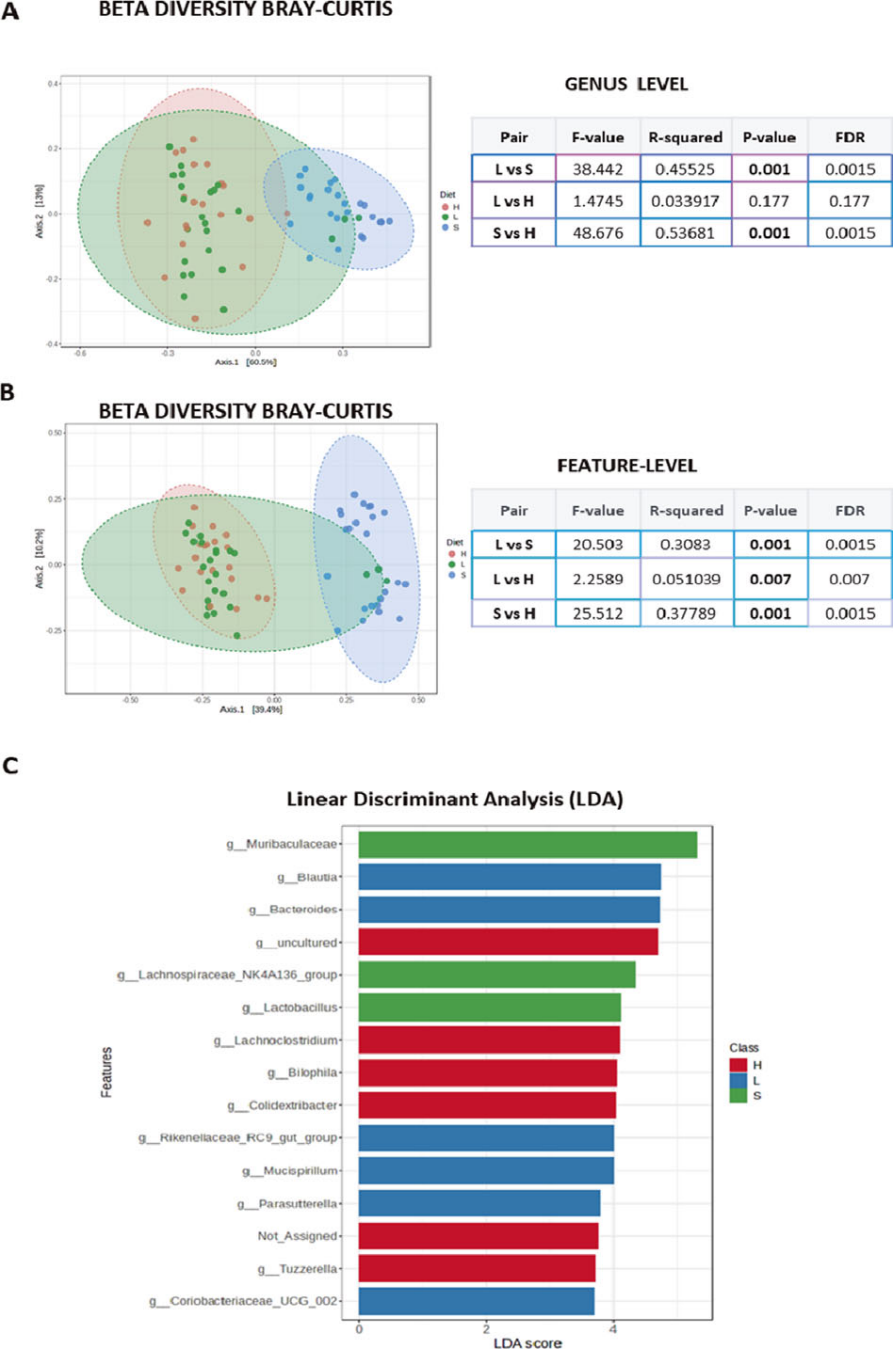
**(A)** Plots show beta diversity analysis from mice groups fed with each diet (S, L, and H), using the Bray–Curtis test at genus taxonomic rank. The corresponding statistical analysis was represented on the right table, representing *p* and DFR values. **(B)** Plots show beta diversity analysis from mice groups feed with each diet (S, L, and H), using Bray–Curtis at feature-level taxonomic rank. In both cases (A, B), the corresponding statistical analysis was represented on the right table, representing *p* and DFR values. (Note that *p* values were represented in bold only when with FDR below 0.04.) **(C)** Plots show linear discrimination analysis (LDA) from mice fed with S, L, and H diets. Data at genus taxonomic rank, show some genera more abundant in each diet, represented when considering LDA >2.

The beta diversity analysis offered a similar scenario, with the comparative analysis at the genus level highlighting clear differences between the **L** and **S** or the **S** and **H** diets (*p* = 0.001 in both cases: Figure 5A). When the feature level was analyzed, the three diets produced significant differences (Figure 5B) and the additional LDA analysis indicated 15 genera were more strongly present in the animals following an **L**, **S**, or **H** diet (e.g., g-*Bilophila*, g-*Lachnoclostridium*, or g-*Tuzzerella*: Figure 5C). The subsequent LEfSe test showed 36 genera that differed significantly, with a very low FDR (<0.03), some of which were very weakly or strongly represented in mice that followed a **H** diet, for instance g-*Prevotellaceae*-UCG001, g-*Ruminococcaceae*, g-*Escherichia_Shigella* or g-*Incertae_Sedis* (Supplementary Figure 11).

Considering that the H diet correlated with an increase in β-amyloid, we tried to identify a signature of this diet through single-factor statistical comparisons using (Differential Expression analysis [DESeq2]). As a result, 24 genera were seen to differ significantly different between mice on an **S** and **H** diet and 4 genera when comparing the **L** and **H** diets. We graphically represent some of the genera that were either increased or decreased when following the **H** diet relative to the **S** and **L** diets (Supplementary Figure 12).

**Figure 6.**
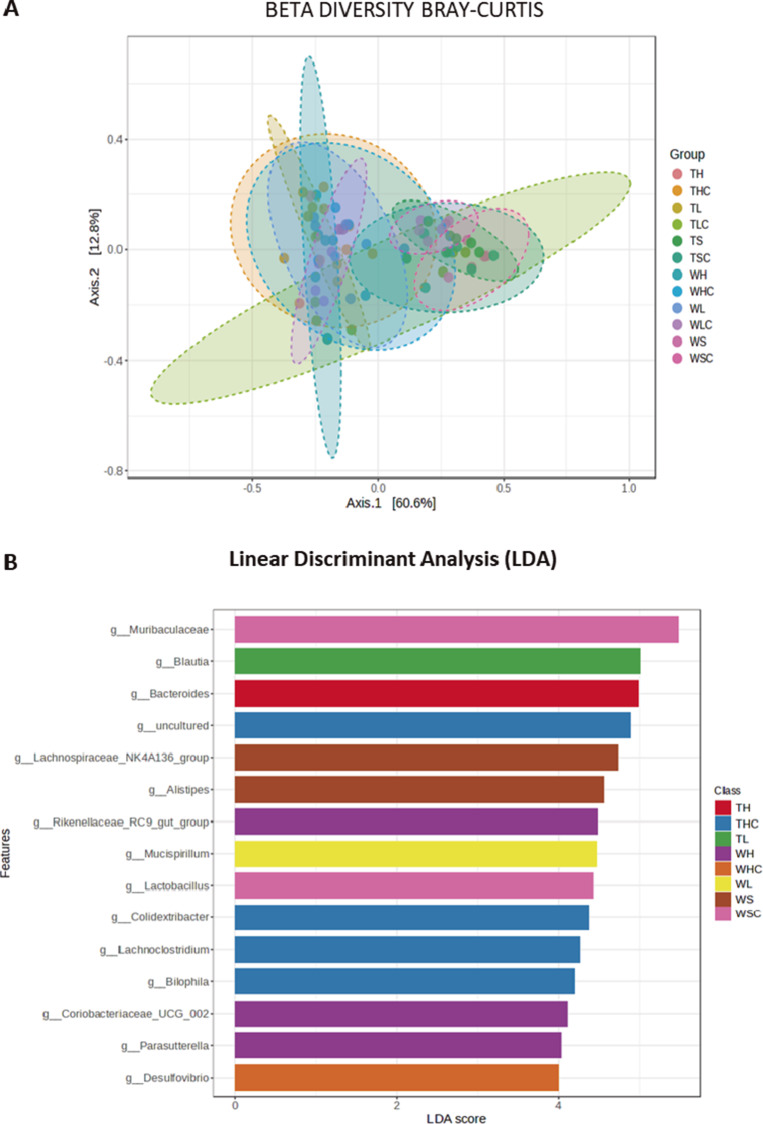
**(A)** Plots show beta diversity analysis from mice groups fed with each diet, when we additionally considered genotype and sexual groups. Considering genotypes, we represented WS, WH, WL; WSC, WHC, WLC (wild-type); and TS, TH, TL, TSC, THC, TLC (APP/PS1), using Bray–Curtis test at genus taxonomic rank. **(B)** Plots show linear discrimination analysis (LDA) from above mice groups at genus taxonomic rank, show some genera more abundant in each diet/genotype, represented when considering LDA >2.

Finally, we analyzed the three variables diet, gonads, and genotype separately, and the data became more complex. The Shanon–Kruskal–Wallis test indicated clear differences were more evident at the genus level where only the comparisons between **TLC** and **TS, WLC** and **WHC,** or **TLC** and **TSC** were not statistically significant (Supplementary Figure 13). Similarly, the beta diversity analysis produced a more complex graphic (Figure 6A) in which all paired comparisons were seen to be significant (Supplementary Figure 14). The LDA test showed some genera were more abundant in certain groups, such as g-*Bacteroides* in **TH** or g-*Bilophila*, g-*Lachnoclostridium*, or *Colidextribacter* in **THC** mice (Figure 6B). Finally, an LEfSe analysis at the genus level showed 42 genera differed significantly when **TG** mice were considered (FDR <0.03**:** Supplementary Figure 15), some of which were higher or lower in the **TG** mice that followed an **H** diet when compared to those on an **L** or **S** diet, such as g-*Prevotellaceae*-UCG001, g-*Lachnoclostridium*, g-*Colidextribacter*, g-*Escherichia_Shigella*, or g-*Incertae_Sedi* (see Supplementary Figure 16).

## Discussion

### GM response in males with different levels of sex hormones

In this study, we first set out to analyze how the GM is modified by the levels of sex hormones in males by comparing males, castrated males, and females for this purpose using a TG AD mouse model and their WT littermates maintained on a standard diet. Initially, WT and TG mice were compared, although the alpha and beta diversity analyses did not show any significant differences between these phenotypes. These homogeneous repertoire of microbiota TG vs WT, contrasts with several reports showing major differences between both genotypes (for review see Zhang et al., 2023). Indeed, in our colony at this age, we only found the g-RF39·genus to be increased in TG mice, using either LEfSe analysis, or the single-factor statistical comparison (DSEeq2), even though this genus represents only 0.12% of total. The RF39 and RFN20 genera are two novel *Tenericutes* lineages of *Bacilli* reported in the gut of humans and domestic animals; however, it remains to be determined whether they are linked to the maintenance of gut homeostasis and microbiome function (Gandy et al., 2019; Wang et al., 2022).

We also analyzed the effect of sex hormones, with the alpha diversity analysis showing clear differences between the **MC** mice and either the **M** or **F** groups at the genus level. By contrast, the beta diversity analysis produced strong differences among the three groups at the phylum and genus levels. The g-*Eubacterium_ventriosum*_group, g-*Muribaculaceae*, and g-*Candidatus_Saccharimonas* g- genera appear to be more strongly represented in **MC** mice. Two phyla and 18 genera appear to be significantly different when analyzed using the LEfSe test. It is interesting to note that in the **MC** mice, the g-*Eubacterium_ventriosum*_group is enhanced relative to the **M** or **F** mice, whereas the g-*Lachnospiraceae*-FCS020 group is almost entirely absent. Some genera are present at an intermediate level in **F** and **M** mice, such as g-*Lactobacillus* or g-*Bacteroides* form, while the g-UCG 009 and g-*Oscillibacter* genera have a similar diversity as in females.

Further support for some of these data was obtained when we analyzed these groups considering the genotypes separately. For instance, g-*E. ventriosum* was more abundant in **WSC** than **WS** or **TSC** than **TS** mice, while g-*Alistipes* was more abundant in **TS** than **TSC**, or **WS** than **WSC** mice. The beta diversity exhibited more significant differences, with the successive LDA test highlighting 15 genera (LDA > 2) and the LEfSe analysis 20 genera that were significantly different.

For example, LDA displayed differences in g *Muribaculaceae*, g-*E. ventriosum*, g-*Candidatus saccharimonas*, g-*Rikenella*, and g-UBA1819 in the **WSC** mice, whereas g-*Alistipes*, g-*Blautia*, and g-*Oscillibacter* were more abundant in **WS** animals. Interestingly, g-*Rikenella* has been associated with degeneration in the APP/PS1 mice model due to a dysregulation of pyrimidine metabolism (Zhang et al., 2017; Feng et al., 2022). However, in our colony, this genus increased in the **FT** mice relative to **FW** mice, although it is not modified in APP/PS1 castrated-male (**TSC**) relative to **TS** mice. This observation did not permit a correlate directly g-*Rikenella* to our APP/PS1 mice, suggesting a sexual hormone effect to be more deeply analysed. Indeed, the single-factor statistical comparison (DSEeq2) confirmed the difference between **WS** and **TS** mice (g-RF39) and those between **WS** and **FS** mice (g-*Lactobacillus*, g-*Clostridia*_UCG_014, and g-*Bacteroides*), in this case perhaps linked to sexual hormones.

In summary, the strong reduction of g-*Alistipes*, g-*Blautia*, and g-*Oscillibacter*, as well as the increase in *Lachnospiraceae*_UCG_001, the g-*Eubacterium-ventriosum* group, or g_*Lactobacillus* was detected in castrated mice, permit to propose that these genera may represent a signature of “testosterone level” that should be analyzed more deeply. In human populations, *Alistipes* have been proposed as protective against certain diseases, including pancreatic cancer, liver fibrosis, and cardiovascular disease. Conversely, *Alistipes* appear to be pathogenic in some diseases like Parkinson′s disease, colorectal cancer, and depression (Parker et al., 2020). The genera *Blautia*, which is a provider of short-chain FAs, and *Alistipes* have been seen to be increased in AD patients (Park et al., 2017; Vogt et al., 2017). These controversial reports strongly suggested that the analysis at the genus level is not enough to have a precise conclusion of their positive/negative effects. Thus, it is tantalizing to propose that g-*Alistipes* and g-*Oscillibacter*, in WT and AD mice, and g-*Blautia* only reduced in WT mice, are responsive to testosterone levels.

Our working hypothesis is that testosterone, may regulate the physiological status of intestine response and the final microbiota/epithelium cross-talk. Finally, this bidirectional cross-talk may be altered by microbiota composition or gastrointestinal tract aging (Baidoo and Sanger, 2024). Indeed, in humans, aging correlated with a sexual hormone reduction (males and females), and this impacts ageing on the intestinal epithelial barrier and immune system (Man et al., 2014). In AD, the intestinal barrier is compromised, which allows gut microbes and molecules to cross the intestinal epithelium (Pellegrini et al., 2023). Thus, it is tantalizing to propose that in our APP/PS1 castrated mice, we are simulating a situation of aging due to the loss of hormones, and the intestinal barrier would be compromised. Working hypothesis that would be further analyzed.

### GM response in three unsaturated ω-3/6 fatty acid diets

The second aim of this study was to determine whether diets with distinct ω-6/ω-3 ratios would modulate an adaptive response of the GM. There is considerable preclinical data from AD and other mouse models that following a high-fat diet generates different GM signatures. Differences in the sensitivity of the GM to dietary modifications have been observed between males and females, specifically using a high-fat diet (Ikemoto et al., 2001) or specific human diets such as ketogenic, Japanese, or Mediterranean diets (Catalan et al., 2002). GM complexity is affected by high-fat intake and sexual differences are present in human populations (Rosser et al., 2022). Thus, our aim was to analyze GM complexity considering “non-fat diets” with a single change in the ω-6/ω-3 ratio, as well as the influence of sex hormones after removing the testes. Thus, we used two modified diets (**H** and **L**, see Methods) that generated final **ω-**6/ω-3 ratios of 3.29 (**L**), 12.17 (**S**), and 25.06 (**H**), while maintaining a similar percentage of total fat (differences of less than 5%). Our previous data showed that in contrast to an **L** or **S** diet, an **H** diet increased the Aβ content, a trend that was enhanced in castrated males albeit not significantly (Ordóñez-Gutiérrez et al., 2021).

Our initial comparison of TG and WT males on each diet using the alpha diversity highlights significant differences. The analysis of relative abundance showed that the phylum *Patescibacteria* was more strongly represented in WT males, and the genera *Alistipes* or Mucispirillum were more abundant in WT mice, in contrast to g-*Muribaculaceae.* The alpha diversity analysis considering the presence or absence of gonads also highlights significant differences, with the phyla *Patescibacteria* and *Deferribacterota* more abundant in **M** as opposed to **MS** mice. In addition, at the genus level, g-Blautia, g-*Alistipes*, and g-Rikenellaceae-RC9 among others were more abundant in **M** mice. The comparative beta analysis of males following all the diets failed to identify significant differences between the **M** and **MC** males (*p* = 057, FDR =0.055). However, at the genus level, the LDA test and LEfSe analysis showed that g-*Alistipes*, g-*Mucispirillum*, g-*Parasutterella*, and g-*Escherichia_Shigella* were significantly more strongly represented in **M** mice, while g-*Romboutsia* and g-*Rikenella* were more significantly present in **MC** mice.

Finally, we analyzed the effect of different diets on the GM in the different groups of mice. The alpha analysis showed that both the **H** and **L** diets generated significant differences relative to the **S** diet at both the phylum or genus levels.

Interestingly, the **H** diet more than the **L** diet, and more than the **S** diet (**H** > **L** > **S**) produced a complex GM pattern, with the phyla *Fermicutes*, *Deferribacterota*, or *Proteobacteria* more abundant in animals that followed the **H** diet. At the genus level, g-Blautia or g-*Alistipes* are more strongly represented in mice following an **H** and **L** diet, whereas g-*Muribaculaceae* are more prevalent in mice following an **S** diet.

As previously mentioned, genera *Blautia* and *Alistipes* have been seen to increase in AD patients (Park et al., 2017; Vogt et al., 2017). In our case, we reported that the H diet increased amyloid burden when compared with the L diet (Ordóñez-Gutiérrez et al., 2021). In addition, the LDA test showed that g-*Muribaculaceae*, g-*Lachnospiraceae*-NK4A136, and g-*Lactobacillus* are more strongly represented in animals following the **S** diet, whereas g-*Colidextribacter*, g-*Bilophila*, g-*Lachnoclostridium*, g-T*uzzerella*, and g-uncultured are positively correlated with the **H** diet. The ensuing LEfSe analysis showed significant differences in 36 genera when the three diets were considered. Thus, in addition to 5 genera previously indicated, g-*Lachnospiraceae*_UCG_006, g-*Anaerotruncus*, g-*Escherichia_Shigella*, g-*Muribaculaceae*, and others are also correlated with following an **H** diet. Some of these differences were confirmed in a single-factor statistical comparison (DESeq2), highlighting significant differences in 29 genera when the **S** and **H** diets are compared and 4 when comparing the **L** and **H** diet. These data reflected two different profiles, one in which these genera increased when the **H** diet was followed (g-lactobacillus and g-Eubacterium_xylanophilum_group) and another in which other genera decreased in abundance (g-*Tuzzerella*, g-*Roseburia*, g-*Lachnoclostridium*, or g-*Escherichia_Shigella*). When we focused only on the **TG** mice fed the three diets, an LEfSe analysis showed significant differences in 42 genera, of which 26 were common to all the groups compared (**TG, WT, MC, M**), whereas some were modified by the **H** diet, such as g-*Prevotellaceae*_UCG001, g-*Lachnoclostridium*, g-*Tuzzerella*, g-*Escherichia_Shigella*, or g-*Lactobacillus.*

The relative abundance of *Firmicutes* is significantly higher when a high-fat diet is maintained (Jo et al., 2021), as seen for *Lactococcus*, *Blautia*, *Lachnoclostridium*, *Oscillibacter*, *Ruminiclostridium*, *Harryflintia*, *Lactobacillus*, *Oscillospira*, and *Erysipelatoclostridium.* Our comparative LEfSe analysis showed some of these genera to be modified in **TH** and **THC** mice, such as g-*Colidextribacter*, g-*Incertae-Sedis*, or g-*Harryflintia*, even though this diet is a low ω-3 diet and not really a high-fat diet. Elsewhere, a higher abundance of pro-inflammatory *Escherichia/Shigella* bacteria has been reported in cognitively impaired older adults without an AD diagnosis (Zhuang et al., 2020). Similarly, an increase in g-*Escherichia/Shigella* and a reduction of *g-Ruminococcaceae* were proposed to be associated with a peripheral inflammatory status of AD patients with cognitive impairment and brain amyloidosis (Cattaneo et al., 2017). Interestingly, data from both genera correlated with our data in mice maintained on aan **H** diet. Moreover, a decrease in the abundance of *L. reuteri* in humans has been correlated with an increase in the incidence of inflammatory diseases (Mu et al., 2018), and *L. reuteri* induces an increase in gut intraepithelial CD4^+^CD8αα^+^ T cells (Cervantes-Barragan et al. 2017). Thus, the decrease in g-Lactobacillus when mice follow an H diet strongly suggests a correlation between dysbiosis and a pathology.

Some controversial data exists regarding g-*Blautia*, and while our **S** and **H** diets strongly increased g-*Blautia*, low levels of *Blautia* have been described in correlation with some pathologies. For instance, g-*Blautia* was negatively correlated with visceral fat accumulation in some human populations, and this genus has the ability to produce bactericins, among other compounds. It was reported that oral administration of *Blautia wexlerae* to mice induces metabolic changes and anti-inflammatory effects that decrease both high-fat-diet-induced obesity and diabetes (Jo et al., 2021), whereas two Blautia species have been associated with visceral fat accumulation (Ozato et al., 2022).

In summary, our data show that the initial mice colony did not present any major differences in GM when maintained on our standard diet, as witnessed when the alpha or beta diversity between TG and WT mice or M and F mice was compared. However, the loss of sex hormones in males generated an important shift in GM diversity, with some genera approximating to that seen in females. A more detailed analysis of these three groups showed the increase/decrease in several genera, and thus, we conclude that these GM adjustments are likely to be associated with sex hormone levels. Accordingly, the levels of sex hormones are something to take into account when studying the impact of diets on human GM populations.

We also analysed the effect of three diets with different ratios of ω-3/ω-6 FAs, and the diet with less ω-3 FAs generated significant changes in GM diversity, affecting more the 40 genera. In addition, we observed some differences between WT and TG mice that followed these diets that suggest additional genetic factors (APP and PS1 mutations in our mouse model) can influence GM diversity. We observed some shifts in genera (increases/decreases) associated with the **H** diet. Considering all the data, it is tantalizing to propose that a combination (reduction/increase) of these genera may be responsible for the intensity of the neuropathology in APP/PS1 mice fed an **H** diet, mostly due to the low levels of ω-3-FAs. These data strongly suggest that a reorganization of the GM and not the modification of one specific genus should be considered responsible for the deterioration of the AD neuropathology in our mouse model.

## Supporting information

Ordoñez-Gutierrez and Wandosell supplementary material Ordoñez-Gutierrez and Wandosell supplementary material

## Supplementary material

The supplementary material for this article can be found at http://doi.org/10.1017/gmb.2025.10005.
