# Supplementary material for: Sex hormones and diets rich in polyunsaturated ω-6/ω-3 fatty acids modify microbiota distinctly in a mouse model of Alzheimer’s disease
Source: Gut Microbiome (Camb). 2025 Jun 18;6:e10. doi: 10.1017/gmb.2025.10005 (PMC12284841; doi:10.1017/gmb.2025.10005)
Supplement: Ordoñez-Gutierrez and Wandosell supplementary material [file S2632289725100054sup001.zip › Diets with HigherFW.pdf]

Sex hormones and diets rich in polyunsaturated ω-6/ω-3 fatty acids modify microbiota distinctly in a mouse model of AD

Lara Ordoñez-Gutierrez [b,c] & Francisco Wandosell [a,c,*]

a. Centro de Biología Molecular "Severo Ochoa" (CSIC-UAM), Calle Nicolas Cabrera 1, Universidad Autónoma de Madrid, 28049 Madrid, Spain
b. Departamento de Bioquímica y Biología Molecular, Plaza de Ramón y Cajal s/n, Universidad Complutense de Madrid, 28040 Madrid Spain
c. Centro de Investigación Biomédica en Red de Enfermedades Neurodegenerativas (CIBERNED), Calle Valderrebollo 5, 28031 Madrid, Spain

* Corresponding author: fwandosell@cbm.csic.es

***Running title**: Sex hormones alter microbiota diversity in an AD mouse model*

ABSTRACT

There is considerable data suggesting that the gut microbiota (GM) contributes to the health of individuals and that when altered, the changes to the microbiota have been linked to metabolic. Moreover, recent evidence suggests that the GM regulates host immunity and influences brain function, findings with implications for neurodegenerative diseases, such as Alzheimer Disease (AD). Thus, GM modulation has become a new paradigm for the clinical management of AD, with efforts focusing on developing novel strategies to prevent or treat this disease. Some of these data are based on dietary modifications, obtained by following a high-fat human diet that is correlated with metabolic disorders. In the present study, using three non-fat diets with different levels of unsaturated ω-6/ω-3 fatty acids (FAs), we first analysed how minor differences in diet can affect the microbiota of APP/PS1 (TG) mice, a model used to study AD. We tailored two diets with two different concentrations of ω-6 and ω-3, generating differences of approx. 6 times differences in ω-3 content. Thus, we used a high ratio ω-6/ω-3 diet (lower amount of ω-3) denoted as H, a low ratio ω-6/ω-3 diet (higher amount of ω-3) denoted as (L), in addition to our standard food (S).

In addition, we studied how the levels of sex hormones may affect the GM by analysing females, castrated and non-castrated males administered the same diet. We used 14 different experimental groups (n=77 mice), and the data were analysed using Microbiome Analyst 2.0 software.

The data obtained show that sex hormones in males fed our standard diet (S) modified alpha and beta diversity, whereas no such differences were observed when TG mice were compared with their wild-type (WT) littermates. Moreover, there were significant differences in both alpha or beta diversity in mice fed with diets with a H or L as opposed to S diet. We previously described that a H diet increases amyloidosis in TG mice. Thus, we try to correlated microbiota changes with this diet.

Here, the comparative analysis of H and L diets highlighted the differences in some microbiota genera, making it tantalizing to suggest that these changes are correlated with the enhanced amyloidosis provoked by a H diet in males.

In conclusion, our data indicate that in a context of a non-high fat diet, the levels of sex hormones or differences in the ω-6/ω-3 FA ratio may alter the GM more than expected. As a consequence, it is tantalizing to propose that low levels of ω-3 FAs in APP/PS1 mice fed an ‘H’ diet may be responsible for modifying some bacterial genera, exacerbating the basal neuropathology in this AD model.

INTRODUCTION

Recently, the gut microbiota (GM) has been shown to play a critical role in human health, in addition to its involvement in the degradation of macronutrients and the production of metabolites (Hooper et al. 2012). Numerous studies indicate that microbial communities are essential factors in many physiological processes including nutrition, inflammation and protection against pathogens (O'Toole and Jeffery 2015)(Ivanov and Honda 2012)(Belkaid and Hand 2014) The GM also produces some key compounds that modulate the innate immune system, affect intestinal epithelial cells and alter endothelial cell function(Silva et al. 2020) generating a barrier that prevents the passage of antigens and bacteria from the gut into the bloodstream (Zhou et al. 2022)(Spadoni et al. 2015). A growing body of clinical and experimental evidence suggests that the composition of the GM may also influence aging and influence brain disorders, such as Alzheimer's Disease (AD)(Verhaar et al. 2022)(Cattaneo et al. 2017), a characteristic of which is the accumulation of amyloid plaques containing the amyloid-β (Aβ) peptide and fibrillary tangles composed of hyperphosphorylated tau. According to the amyloidogenic theory, the accumulation of Aβ-polymers induces oxidative stress, mitochondrial dysfunction and synaptic impairment, provoking a glial response and neurodegeneration, and ultimately impairing cognitive performance (Hardy and Selkoe 2002)(Selkoe 2002)(Hunag et al. 2016). A small percentage of AD cases have a genetic origin, Familial AD (FAD) that is caused by mutations in the Amyloid Precursor Protein (APP)(Mullan et al. 1992) , Presenilin 1 and 2genes (Levy-Lahad et al. 1995)(Sherrington 1996). However, more than 98% of AD cases are sporadic (of unknown cause). A GWAS analysis of different sporadic cohorts highlighted more than 65 risk factors, including several cellular and metabolic elements that may be involved in AD (Bellenguez et al. 2022). Indeed, aging, environmental and/or dietary factors may have a strong influence on the appearance of this disease, and increasing evidence suggests an association between AD and certain metabolic disorders, including diabetes or hypercholesterolemia, which are also considered as risk factors (Ettcheto et al. 2015)(Park et al. 2013)(Ricciarelli et al. 2012) .

There is some clinical and preclinical evidence supporting an involvement of the GM in promoting AD onset and progression. For example, amyloid deposition has been associated with the presence of pro-inflammatory bacterial species in the gut and pro-inflammatory cytokines in the blood(Vogt et al. 2017)(Zhuang et al. 2018). Preclinical studies showed that elements of the GM are necessary for brain amyloid deposition (Liu et al. 2019) and that the GM composition may be associated with neurodegeneration (Verhaar et al. 2022)(Sheng et al. 2022). Significantly, antibiotic-mediated perturbations in the GM were proposed to modulate amyloid deposition in an AD mouse model (Minter et al. 2016), and an association between brain amyloidosis and pro-inflammatory gut bacteria was highlighted in cognitively impaired patients (Fishbein et al. 2023) (Cattaneo et al. 2017)(Munir et al. 2024). Some data suggests that the GM may influence brain physiology through signalling driven by the GM in this so-called microbiota-gut-brain axis (MGBA) (Frost et al. 2014)(Vernocchi et al. 2016)(Munir et al. 2024). Obviously, diet is an important element

that can affect the GM and there is some evidence that specific diets may be correlated with AD (Scarmeas et al. 2006)(Morris et al. 2003). Particular attention has focused on two major classes of polyunsaturated fatty acids (PUFAs), the omega-3 and omega-6 (ω-3/6) FAs (Sambra et al. 2021)(Burlingame et al. 2009). Indeed, there are reports of a clear decrease in the risk of suffering AD and other dementias in people who consume large quantities of fish containing high levels of ω-3/6 PUFAs (Morris et al. 2003) (Crawford and Broadhurst 2012). The direct relationship between ω-3 FAs in the diet, mostly Docosahexaenoic acid (DHA-22:6 ω-3), and the evolution of a neuro-degenerative pathology has been addressed in animal models (Hooijmans et al. 2007)(Herrera et al. 2019)(Cederholm and Palmblad 2010). Consequently, lower ω-3 FA levels in the diet have been linked to an increased risk of developing neurodegenerative diseases in rodent models and in humans (Barron et al. 2013)(Ikemoto et al. 2001)(Catalan et al. 2002). However, some clinical trials using ω-3 FAs in humans failed to establish a clear link (Barberger-Gateau 2002).Another important consideration is the effect of gender reported. Even though a diet supplemented with DHA may reduce the hallmarks of AD in mouse models (Calon et al. 2004)(Green et al. 2007)(Perez et al. 2010), the presence of sex hormones may modify the final effect of DHA in some AD models at least. We previously found that high levels of DHA [22:6 ω-3] in the diet were not directly correlated with high levels of DHA in the brain cortex, both in male and female mice. However, the levels of sex hormones (oestrogen or testosterone) influence the effect of a DHA rich diet on amyloid burden (Herrera et al. 2018)(Ordóñez-Gutiérrez et al. 2021). These data raise some questions into the effect of ω-6/ω-3 rich diets on the GM and on how ω-6 and ω-3 FAs modulate GM populations. Another question is how gender may influence the effect of a ω-6/ω-3 rich diet, particularly given that human aging is strongly associated with AD and with a physiological reduction in the levels of sex hormones

As a result, a double-transgenic APPswe/PS1ΔE9 mouse AD model (TG) and their wild-type (WT) littermates were used here to determine how the GM is modified by changes in the levels of sex hormones, comparing male, castrated male and female mice. In addition, the influence of diet on these alterations to the GM were assessed in animals fed a standard (**S**) diet or a low (**L)** or high (**H**) ω-6/ω-3 ratio diet. The data obtained show that the sex hormones do not appear to modify alpha and beta diversity in males following a **S** diet. However, when mice followed a **H** or **L** diet there were significant differences in alpha and beta diversity relative to those following a **S** diet. This comparative analysis makes it tantalizing to propose that following a **H** diet enhances amyloidosis in males.

Materials and Methods

Animals and Husbandry

APP/PS1 TG mice were purchased from Jackson Laboratories (Bar Harbor; stock no. 005864), the B6.Cg-Tg [APPSwe, PSEN1dE9] 85Dbo/J strain overexpressing the human APP gene carrying the Swedish mutation and exon-9-deleted PSEN1 (Jankowsky et al. 2001). The mice were housed at constant temperature (22 ± 2 °C) and humidity (50 ± 5%), and on a 12:12 h light–dark cycle in a specific-pathogen-free animal facility. The orchiectomy procedure was performed following standard protocols (Sophocleous and Idris 2019). All animal care and handling complied strictly with current Spanish legislation and guidelines, and those of the European Commission [directive 2010/63/EU]. All the procedures involving the use and management of the TG mouse colony were approved by the Spanish Research Council (CEEA-CBMSO-33/307), the Community of Madrid (PROEX 341/15, recently extended by 5 years, and Ref. PROEX 069.7/21), and the Spanish Research Council [CEEA-CBMSO-23/307.1].

Genotyping

The genotype of the pups was confirmed by PCR analysis (Ordóñez-Gutiérrez et al. 2015) using three primers: one anti-sense primer matching the sequence in PrP (5′ GTG GAT ACC CCC TCC CCC AGC CTA GAC C); one transgene specific sense primer (PS1 5′ CAG GTG GTG GAG CAA GAT G; APP 5′ CCG AGA TCT CTG AAG TGA AGA TGG ATG), and a second sense specific primer for genomic PrP (5′ CCT CTT TGT GAC TAT GTG GAC TGA TGT CGG). Only one prion (PrP) gene band, used as internal control, was detected in the WT mice, whereas three bands (APP, PS1 and PrP) were observed in the TG mice.

Diets

Two diets were prepared by Up-Science [France] and in addition, our standard diet (**S**) was analysed by the same company to determine the final percentage of each lipid (Supplementary Table 1A**)**. Both the TG and WT genotypes used in these experiments (males, castrated-males and females) were fed these diets for 90 days, from 3 to 6 months of age. In all cases the mice were allowed free access to food and tap water *ad libitum*. The experiments began from 3 months post-partum and the animal's body weight was measured weekly. The **S** diet was gradually replaced by the corresponding experimental diet at the outset of the experiments (see below), the latter maintained until the animals reached 6 months of age (endpoint) when they were then sacrificed by CO_2 inhalation.

-Standard food (**S**), as used in our animal facility, was purchased from Scientific Animal Food and Engineering (France), and it had a ω-6/ω-3 ratio of 12:3.

-The H diet (**H**), with a higher ω-6/ω-3 ratio, is an example of a very low ω-3 PUFA content diet. This pellet diet contains 34.5% ω-6 PUFAs and 1.4% ω-3 PUFAs, with a resulting ω-6/ω-3 ratio of 24:6.

-The L diet (**L**), had a lower ω-6/ω-3 ratio, in this case with fish-derived ω-3 PUFAs, and with 30.2% ω-6 and 9.4% ω-3 PUFAs resulting in a ω-6/ω-3 ratio of 3:2.

None of these diets can be considered a “high-fat diet” as the total lipid content was similar to that of the **S** diet fat content. Total fat content: **L** 69 g/kg; **S** 46 g/kg; and **H** 73 g/kg (Ordóñez-Gutiérrez et al. 2021) .

Metagenomics

The metagenomics analysis was performed at the CBMSO Genomics and NGS Core Facility (GENGS) services, and the data was analysed with assistance from the Centro de Computación Científica - Universidad Autónoma de Madrid (CCC-UAM, https://www.ccc.uam.es/). The metagenomics experiment set out to define the GM in the 14 different experimental groups of mice, contemplating different sex hormone status, genotype (WT or TG mice); and Standard and High/Low DHA content diets (**S, H** and **L,** respectively**)**. The experimental groups are as follows:

**General characteristics of the experimental groups**				
**Group**	**Sex**	**Genotype**	**Diet**	**# Animals**
WH	Male	WT	High-DHA	5
WL	Male	WT	Low-DHA	6
WS	Male	WT	Standard	6
TH	Male	TG	High-DHA	3
TL	Male	TG	Low-DHA	6
TS	Male	TG	Standard	6
WHC	Male-castrated	WT	High-DHA	6
WLC	Male-castrated	WT	Low-DHA	6
WSC	Male-castrated	WT	Standard	5
THC	Male-castrated	TG	High-DHA	6
TLC	Male-castrated	TG	Low-DHA	6
TSC	Male-castrated	TG	Standard	7
FW	Female	WT	Standard	5
FT	Female	TG	Standard	4

DNA isolation and 16S gene sequencing

DNA was extracted using the *DNeasy PowerLyzer* DNA isolation kit (Qiagen, Hilden, Germany) according to the manufacturer’s instructions. Bacterial gut biodiversity in these mice was assessed by amplifying the 16S region. The “complete sequence” includes the tails needed for Illumina indexing and sequence inclusion, and the “amplified sequence” includes the nucleotides to be trimmed from reads. The characteristics of the primers used are shown in (Supplementary Table 1B).

The data was analysed using the MicrobiomeAnalyst 2.0 software, specifically Qiime2 software5 was used for the metagenomics analysis, which is an open-source bioinformatics package to analyse the microbiome from raw DNA sequencing data (https://cutadapt.readthedocs.io/en/stable/). We used the q2-dada2 plugin.

DADA2 is a complete pipeline to detect and correct Illumina amplicon sequence data, joining the reads, and to filter chimeric sequences (https://github.com/neufeld/pandaseq).

Data Availability

The sequencing data presented in this study can be found in the European Nucleotide Archive (ENA) online repository, accession number (# Study PRJEB79953) [https://www.ebi.ac.uk/ena/].

The data was organized into two sets:

- Subset 1 (**S Diet**): mice on a standard diet. This subset will allow the differences in the GM to be defined depending on sex/circulating hormones and genotype under normal metabolic status, as the diet is unmodified.

- Subset 2 (**Males**): males only. This subset will allow the effect of altering diet on the GM to be determined, as well as the effect of the presence/absence of gonads and the genotype in one gender.

To compare the GM composition between the groups, we first used α-diversity and β-diversity parameters, using the rarefied microbiota data to calculate the alpha and beta diversity. As a general rule we used "phylum level" and "genus level" as the standard analysis, and only in few cases "feature-level" was represented.

A qualitative and statistical analysis of subsets 1 and 2 were performed with the MicrobiomeAnalyst 2.0 web software (https://www.microbiomeanalyst.ca/). The α-diversity parameter is evaluated at the feature (OTU/ASV) level using Shannon, and significant differences are evaluated using Mann-Whitney/Kruskal-Wallis, (Posthoc) pairwise comparisons, test for continuous variables with non-normal distributions.

Beta-diversity:.For beta-diversity analysis we used PCoA plot. By default, the difference in diversity is assessed using the Bray–Curtis index. When comparing two (or more) groups, we used a permutational multivariate statistical analysis of variance (PERMANOVA)(Pairwise-PERMANOVA). We used this route: BIOM format and QIIME v1.5.0. Dta filtering: A 20% prevalence filter means at least 20% of its values should contain at least 4 counts; and a Low variance filter of 10% was applied, using inter-quantile range (IQR). The normalization consists of the following options:1. Data rarefying was performed by randomly subsampling without replacement to the size of the smallest library that is not considered defective"2. Data scaling: Total sum scaling (TSS), Cumulative sum scaling (CSS), Upper-quantile normalization (UQ). 3. Data transformation: Relative log expression (RLE), Trimmed mean of M-values (TMM), Centred log ratio (CLR). Rarefaction was performed with all samples. Performed total sum normalization. No data transformation was performed.

In addition, we used Microbiome Analyst software to establish Principal Components plot, the Linear Discriminant Analysis (LDA, scores ≥2) and the Linear Discriminant Analysis Effect Size (LEfSe), as well as a Single-factor Statistical Comparisons (DESeq2) to identify the specific biomarker group. This analysis focuses on both the statistical significance and biological relevance, helping identify biomarkers that exhibit

substantial differences between the groups. A Mann-Whitney test was used for single-factor statistical comparisons, and the Kruskal-Wallis test was used to assess whether 2 or more samples come from the same distribution. The abundance of the most significantly altered genus among the experimental groups was represented as log-transformed values, derived from the LEfSe or Single-factor Statistical comparisons data [False Discovery Rate (FDR) <0.05]

.

RESULTS

Comparative analysis of the microbiota in WT and TG mice fed a standard (S) diet

The GM data from WT and TG mice was analysed using the Shannon test to address variability in metagenomics samples, a test that considers richness and distribution [evenness], while the beta diversity was analysed with the Bray-Curtis Dissimilarity test. Our first aim was to determine whether differences in the GM could be detected between TG and WT mice fed the standard diet used at our animal facility (**S**), analysing samples obtained from male and female mice of both genotypes (See all schematic representation in Figure 1A). The alpha analysis showed a similar level of complexity between WT and TG mice, with no significant differences at the phylum or genus level (Supplementary Figure 1A). The relative abundance anlysis showed that Patescibacteria were more abundant in WT samples (Supplementary Figure 1B), while at the genus level Alistipes or Desulfovibrio were more abundant in the TG samples (Supplementary Figure 1B).

When the beta diversity was assessed between both genotypes, no major differences were detected at the phylum level (Permanova test, p= 0.787,) or at the genus level (Permanova test, p= 0.494; Supplementary Figure 1C).

Subsequently, the LEfSe was used to identify genomic features that differed significantly between each group in our metagenomic analyses (i.e., phylum, genus and species). At the family and genus level, only g-RF39 was more strongly represented in TG samples relative to the WT male mice (p=3.96E-4: Supplementary Figure 1D), as further confirmed using a Single-factor Statistical Comparison (Mann-Whitney/Kruskal-Wallis test, p=4.31E-4; Supplementary Figure 1E).

Sex hormones and diversity

Within the group maintained on a **S** diet, we determined whether different levels of sex hormones (testostorene and derivatives) might affect the GM. Three groups of animals were established, females (**F**), males (**M**) and castrated males (**MC**) (Scheme Figure 1A) and when these groups were considered, the Shannon index failed to detect significant differences among the groups at the phylum level. By contrast, differences between **MC**, and **M** or **F** were evident at the genus level (p=0.044902 and 0.049073 respectively, Kruskal-Wallis: Figure 1B). By analysing the relative abundance in these groups, at the phylum level Proteobacteria were more abundant in **F** than in **M** mice, and more than in **MC** mice (**F>M>MC**: Supplementary Figure 2A). At the genus level, we detected clear differences and for instance, g-Bacteroides and g-Lactobacillus were more abundant in **F** than in **MC** mice, and lower in **M** (**F>MC>M**). By contrast, some genera were more abundant in **M**, such as g-Alistipes or g-Blautia, and they were clearly reduced in **F** and **MC** mice (Figure 1B).

The analysis of beta diversity failed to identify significant differences at the phylum level (Supplementary Figure 2B), whereas the three groups presented differences at the genus level (Permanova test - **F** vs **MC**, p-value 0.005, **F** vs **M**, p-value 0.001; **MC** vs **M**, p-value 0.009) and at the feature level (Permanova test - **F** vs **MC**, p-value 0.002; **F** vs **M,** p-value 0.001; **MC** vs **M**, p-value 0.001: Figure 2B).

Through a LEfSe analysis we found a significant increase in p-Proteobacteria in **F** and p-Patescibacteria in **MC** at the phylum level (Figure 2B), whereas several differences were observed at the genus level associated with the gonads, such as those in g-Blautia in **M**, g-Lactobacillus in **F**, or g-Candidatus Saccharimonas in **MC** (Figure 2C and Supplementary Figure 2C). At the species level, the data confirmed the increase in the presence of Bacteroides *caecimuris* and Lactobacillus *reuteri* in **F** mice, which might represent a "female mouse signature" (Supplementary Figure 3).

Comparative analysis of microbiota between WT and TG mice following a standard diet and the effect of sex hormones

The effect of different sex hormones

To extend our data on the effect of diet on the GM, we set out to assess the influence of sex hormones by considering six experimental groups: WT females (**FW**) and TG females (**FT**), WT (**WS**) and TG (**TS**) male mice and, and WT (**WSC**) and TG (**TSC**) castrated male mice. An alpha analysis identified some significant differences at the genus and species levels, although we only considered the differences between **WS** and **WSC** to be particularly relevant (p=0.00433, FDR = 0.064: Figure 3A, Supplementary Figure 4A). Histograms highlighted the alterations in GM composition, with a reduction in the phylum Proteobacteria in **WSC** and **TSC** mice (Supplementary Figure 4B). At the genus level there were also some clear differences, for instance there was a reduction in Alistipes in **TSC** and **WSC** mice when compared with **TS** relative or **WS** respectively. The genus Bacteroides was more abundant in female TG animals (**FT>FW**) and in castrated males (**TSC** and **WSC**), whereas Oscillibacter was more abundant in males (**TS** and **WS**) than in females (**FW** and **FT**: Figure3B).

The beta diversity analysis showed no major differences at the phylum level but significant differences at the genus level, for instance when comparing **WS** with either **WSC** (p-value= 0.006) or against **FW** and **FT** (p-value= 0.005 and 0.006, respectively**:** Supplementary Figure 5A). When a Bray-Curtis comparison was performed at the group feature level, almost all groups were significantly different except for **FT** vs **FW** or **WSC** vs **TSC** (Figure 3C).

A Linear Discriminant Analysis at the genus level identified differences in g-Blautia, g-Alistipes and g-Oscillibacter in **WS** mice, or in g-Rikenella, g-Candidatus Saccharimonas and g-UBA1819 in **WSC** mice, among others (Supplementary Figure 5B). A more extensive LEfSe analysis showed a significant increase in

p-Proteobacteria in **FT** mice and p-Patescibacteria in **WSC** mice at the phylum level, in accordance with the previous data (Supplementary Figure 6). Moreover, at genus level 20 significantly different changes were identified (Supplementary Figure 6), including those to g-Blautia, g-Alistipes, g-Lactobacilus. Some specific analyses were performed using Single-factor Statistical Comparisons (EdgeR), showing significant differences in genera between **WS** and **FW** mice (i.e., g-Lactobacillus or g-Clostridia UCG014), or **WS** and **TS** mice (g-RF39 or g-Eubacterium_brachy_group), as well as an effect of testosterone depletion in **WS** vs **WSC** mice (i.e., g-Lactobacillus or g-Peptococcus or g-Eubacterium ventriosum group: Supplementary Figure 7).

The effect of diets containing different amount of unsaturated ω-3/6 fatty acids

The second aim of this work was to analyse the differential effects of three diets that had a similar total fat composition but with different levels of ω-6/ω-3 PUFAs. We previously reported that these diets modified the amyloid burden in APPSWE/PS1ΔE9 male and female mice. Hence, here we studied the populations of mice considered above (males, castrated males and female mice of both genotypes -**WT** and **TG**) when they were fed with these diets: standard diet (**S**), low (**L**) and high (**H**) ω-6/ω-3 ratio diets. We first performed an alpha analysis that took into account both genotypes, which highlighted some differences in GM abundance. Significant differences were evident at the phylum level (**WT** vs **TG**, p=0.014), for instance with a clear reduction of p-Patescibacteria (Supplementary Figure 8A-B). Likewise, at the genus level g-Muribaculaceae was more abundant in **TG** mice, whereas g-Alistipes and g-Mucispirillum were present in greater quantities in **WT** mice (Supplementary Figure 8C). Similarly, the alpha analysis of castrated versus non-castrated males showed statistically differences at both the phylum and genus levels (Supplementary Figure 9A). In terms of relative abundance, there was a clear reduction in p-Patescibacteria and p-Deferribacterota in **MC** mice, whereas at the genus level, g-Muribaculaceae was more abundant in **MC** mice, whereas g-Blautia, g-Alistipes, g-Rikenellaceae-RC90 or g-Mucispirillum were more abundant in **M** mice (Supplementary Figure 9B-C).

In terms of beta diversity, no major differences were evident at the phylum or genus level between **WT** and **TG** mice (Supplementary Figure 10A), whereas there were significant differences at the feature-level between **MC** and **M** mice (p=0.004), and almost significant differences at the genus level (p=0.057, FDR =0.057: Supplementary Figure 10B). The subsequent LDA analysis showed the genera g-Romboutsia and g-Rikenella were more abundant in **MC** mice, also remaining as the most abundant genera in **M** mice (Supplementary Figure 10C). The additional LEfSe statistics showed that p-Proteobacteria and p-Deferribacterota were significantly more abundant in **M** mice at the phylum level, and 10 genera were significantly different, with g-Rikenella and g-Romboutsia most abundant in **MC** mice (Supplementary Figure 10D).

An alpha analysis was then performed that focused on the three diets, and the data showed, at phylum level, that the **S** diet had a significant effect relative to both the **L** and **H** diet (S vs L- 3.1 E-7; S vs H- 3.089E-10), with no significant differences between the **L** and **H** diet(Figure 4A,left panel)..

At genus level, the differences between the **S** and **L** or the **S** and **H** diets were very significant (S vs L- 3.82 E-8; S vs H- 7.609 E-11) in contrast to those between the **H** and **L** diet (Figure 4A, right panel**).** The analysis of relative abundance showed that p-Bacteroidota was more abundant in mice following the **S** diet, whereas p-Firmicutes, p-Deferribacterota and p-Proteobacteria more abundant in the animals following a **H** or **L** diet (Figure 4A-B). Differences were evident at the genus level, for instance in the greater abundance in g-Blautia and g-Lactobacillus in animals following a **H** or **L** diet**.** As a general rule, both the mice following a **H** and **L** diet had a wider distribution of some genera due to the relative reduction of g-Muribaculaceae, which was more abundant in those following a **S** diet (Figure 4B-C).

The beta diversity analysis offered a similar scenario, with the comparative analysis at the genus level highlighting clear differences between the **L** and **S** or the **S** and **H** diets (p= 0.001 in both cases: Figure 5A). When the feature level was analysed, the three diets produced significant differences (Figure 5B) and the additional LDA analysis indicated 15 genera were more strongly present in the animals following a **L**, **S** or **H** diet (e.g., g-Bilophila, g-Lachnoclostridium or g-Tuzzerella: Figure 5C). The subsequent LEfSe test showed 36 genera that differed significantly, with a very low FDR (<0.03), some of which were very weakly or strongly represented in mice that followed a **H** diet, for instance g-Prevotellaceae-UCG001, g-Ruminococcaceae, g-Escherichia_Shigella or g-Incertae_Sedis (Supplementary Figure 11).

Considering that the H diet correlated with an increase in β-amyloid, we tried to identify a signature of this diet through Single-factor Statistical Comparisons using (DESeq2). As a result, 24 genera were seen to differ significantly different between mice on a **S** and **H** diet, and 4 genera when comparing the **L** and **H** diets. We graphically represent some of the genus that were either increased or decreased when following the **H** diet relative to the **S** and **L** diets (Supplementary Figure 12).

Finally, we analysed the three variables diet, gonads and genotype separately, and the data became more complex. The Shanon-Kluskal-Wallis test indicated clear differences were more evident at the genus level where only the comparisons between **TLC** and **TS, WLC** and **WHC** or **TLC** and **TSC** were not statistically significant (Supplementary Figure 13**).** Similarly, the beta diversity analysis produced a more complex graphic (Figure 6A) in which all paired comparisons were seen to be significant (Supplementary Figure 14). The LDA test showed some genera were more abundant in certain groups, such as g-Bacteroides in **TH**, or g-Bilophila, g-Lachnoclostridium or Colidextribacter in **THC** mice (Figure 6 B). Finally, a LEfSe analysis at the genus level showed 42 genera differed significantly when **TG** mice were considered (FDR <0.03**:** Supplementary Figure 15), some of which were higher or lower in the **TG** mice that followed a **H** diet when

compared to those on a **L** or **S** diet, such as g-Prevotellaceae-UCG001, g-Lachnoclostridium, g-Colidextribacter, g-Escherichia_Shigella or g-Incertae_Sedi (see Supplementary Figure 16).

Discussion

GM response in males with different levels of sex hormones

In this study, we first set out to analyse how the GM is modified by the levels of sex hormones in males by comparing males, castrated males and females, for this purpose using a TG AD mouse model and their WT littermates maintained on a standard diet. Initially, WT and TG mice were compared, although the alpha and beta diversity analyses did not show any significant differences between these phenotypes. These homogeneous repertoire of microbiota TG vs WT, contrast with several with several reports showing major differences between both genotypes (for review see Zang et al.,2023). Indeed, in our colony at this age, we only found the g-RF39·genus to be increased in TG mice, using either LEfSe analysis , or the single-factor statistical comparison (DSEeq2), even though this genus represent only 0.12% of total .The RF39 and RFN20 genera are two novel Tenericutes lineages of Bacilli reported in the gut of humans and domestic animals, however it remains to be determined whether they are linked to the maintenance of gut homeostasis and microbiome function (Gandy et al., 2019) (Wang et al., 2020).

We also analysed the effect of sex hormones, with the alpha diversity analysis showing clear differences between the **MC** mice and either the **M** or **F** groups at the genus level. By contrast, the beta diversity analysis produced strong differences among the three groups at the phylum and genus level. The g-Eubacterium_ventriosum_group, g-Muribaculaceae, and g-Candidatus_Saccharimonas g- genera appear to be more strongly represented in **MC** mice. Two phyla and 18 genera appear to be significantly different when analysed using the LEfSe test. It is interesting to note that in the **MC** mice, the g-Eubacterium_ventriosum_group is enhanced relative to the **M** or **F** mice, whereas the g-Lachnospiraceae-FCS020 group is almost entirely absent. Some genera are present at an intermediate level in **F** and **M** mice, such as g-Lactobacillus or g-Bacteroides from, while the g-UCG 009 and g-Oscillibacter genera have a similar diversity as in females.

Further support for some of these data was obtained when we analysed these groups considering the genotypes separately. For instance, g-Eubacterium ventriosum was more abundant in **WSC** than **WS** or **TSC** than **TS** mice, while g-Alistipes was more abundant in **TS** than **TSC**, or **WS** than **WSC** mice. The beta diversity exhibited more significant differences, with the successive LDA test highlighting 15 genera (LDA>2) and the LEfSe analysis 20 genera that were significantly different.

For example, LDA displayed differences in g Muribaculaceae, g-Eubacterium ventriosum, g-Candidatus saccharimonas, g-Rikenella and g-UBA1819 in the **WSC** mice, whereas g-Alistipes, g-Blautia and g-

Oscillibacter were more abundant in **WS** animals. Interestingly, g-Rikenella has been associated with degeneration in the APP/PS1 mice model due to a dysregulation of pyrimidine metabolism (Feng et al. 2022) (Zhang et al. 2017). However, in our colony this genus increased in the **FT** mice relative to **FW** mice, although it is not modified in APP/PS1 castrated-male (**TSC**) relative to **TS** mice. This observation did not permit correlate directly g-Rikenella to our APP/PS1 mice, suggesting a sexual hormone effect to be more deeply analysed. Indeed, the single-factor statistical comparison (DSEeq2) confirmed the difference between **WS** and **TS** mice (g-RF39) and those between **WS** and **FS** mice (g-Lactobacillus, g-Clostridia_UCG_014 and g-Bacteroides), in this case perhaps linked to sexual hormones.

In summary the strong reduction of g-Alistipes, g-Blautia and g-Oscillibacter, as well as the increase in Lachnospiraceae_UCG_001, the g-Eubacterium-ventriosum group, or g_Lactobacillus was detected in castrated mice, permit to propose that these genera may represent a signature of "testosterone level" that should be analysed more deeply. In human populations, Alistipes have been proposed as protective against certain diseases, including pancreatic cancer, liver fibrosis and cardiovascular disease. Conversely, Alistipes appear to be pathogenic in some diseases like Parkinson's disease, colorectal cancer and depression (Parker et al. 2020). The genera Blautia , which is a provider of short chain fatty acids, and Alistipes have been seen to be increased in AD patients (Park et al , 2017)(Vogt et al., 2017) . These controversial reports strongly suggested that the analysis at genus level is not enough to have a precise conclusion of their positive/negative effects. Thus, it is tantalizing to propose that g-Alistipes, and g-Oscillibacter, in WT and AD mice, and g-Blautia only reduced in WT mice, are responsive to testosterone levels.

Our working hypothesis is that testosterone, may regulate the physiological status of intestine response, and the final microbiota/epithelium cross-talk. Finally, this bidirectional cross-talk may be altered my microbiota composition or gastrointestinal tract aging (Baidoo N. &. SangerGJ., 2024). Indeed, in human, aging correlated with a sexual hormones reduction (males and females) and this impact in ageing on the intestinal epithelial barrier and immune system (Man at al., 20114). In AD, the intestinal barrier is compromised, which allows gut microbes and molecules to cross the intestinal epithelium (Pellegrini et al., 2023). Thus, it is tantalizing to propose that in our APP/PS1 castrated mice we are simulating a situation of aging due to the loss of hormones, and the intestinal barrier would be compromised. Working hypothesis that would be further analysed.

GM response in three unsaturated ω-3/6 fatty acid diets

The second aim of this study was to determine whether diets with distinct ω-6/ω-3 ratios would modulate an adaptive response of the GM. There is considerable preclinical data from AD and other mouse models that following a high-fat diet generates different GM signatures. Differences in the sensitivity of the GM to dietary modifications have been observed between males and females, specifically using a high fat diet

(Ikemoto et al. 2001) or specific human diets such as ketogenic, Japanese or Mediterranean diets (Catalan et al. 2002). GM complexity is affected by high-fat intake and sexual differences are present in human populations (Rosser et al. 2022). Thus, our aim was to analyse GM complexity considering "non-fat diets" with a single change in the ω-6/ω-3 ratio, as well as the influence of sex hormones after removing the testes. Thus, we used two modified diets (**H** and **L**, see Methods) that generated final **ω-**6/ω-3 ratios of 3.29 (**L**), 12.17 (**S**) and 25.06 (**H**), while maintaining a similar percentage of total fat (differences of less than 5%). Our previous data showed that in contrast to a **L** or **S** diet, a **H** diet increased the Aβ content, a trend that was enhanced in castrated males albeit not significantly (Ordóñez-Gutiérrez et al. 2021).

Our initial comparison of TG and WT males on each diet using the alpha diversity highlights significant differences. The analysis of relative abundance showed that the phylum Patescibacteria was more strongly represented in WT males, and the genera Alistipes or Mucispirillum were more abundant in WT mice, in contrast to g-Muribaculaceae. The alpha diversity analysis considering the presence or absence of gonads also highlights significant differences, with the phyla Patescibacteria and Deferribacterota more abundant in **M** as opposed to **MS** mice. In addition, at the genus level, g-Blautia, g-Alistipes and g-Rikenellaceae-RC9 among others were more abundant in **M** mice. The comparative beta analysis of males following all the diets failed to identify significant differences between the **M** and **MC** males (p=057, FDR =0.055). However, at genus level the LDA test and LEfSe analysis showed that g-Alistipes, g-Mucispirillum, g-Parasutterella and g-Escherichia_Shigella were significantly more strongly represented in **M** mice, while g-Romboutsia and g-Rikenella were more significantly present in **MC** mice.

Finally, we analysed the effect of different diets on the GM in the different groups of mice. The alpha analysis showed that both the **H** and **L** diets generated significant differences relative to the **S** diet at both the phylum or genus level.

Interestingly, the **H** diet more than the **L** diet, and more than the **S** diet (**H**>**L**>**S**), produced a complex GM pattern, with the phyla Fermicutes, Deferribacterota or Proteobactaria more abundant in animals that followed the **H** diet. At the genus level, g-Blautia or g-Alistipes are more strongly represented in mice following a **H** and **L** diet, whereas g-Muribaculaceae are more prevalent in mice following a **S** diet.
As previously mentioned genera Blautia and Alistipes have been seen increased in AD patients (Park et al , 2017)(Vogt et al., 2017) . In our case we reporter that H diet increased amyloid burden when compared with L diet (Ordóñez-Gutiérrez et al. 2021). In addition, the LDA test showed that g-Muribaculaceae, g-Lachnospiraceae-NK4A136 and g-Lactobacillus are more strongly represented in animals following the **S** diet, whereas g-Colidextribacter, g-Bilophila, g-Lachnoclostridium, g-Tuzzerella and g-uncultured are positively correlated with the **H** diet. The ensuing LEfSe analysis showed significant differences in 36 genera when the three diets are considered. Thus, in addition to 5 genera previously indicated g-Lachnospiraceae_UCG_006, g-Anaerotruncus, g-Escherichia_Shigella, g-Muribaculaceae and others are

also correlated with following a **H** diet. Some of these differences were confirmed in a single-factor statistical comparison (DESeq2), highlighting significant differences in 29 genera when the **S** and **H** diets are compared, and 4 when comparing the **L** and **H** diet. These data reflected two different profiles, one in which these genera increased when the **H** diet was followed (g-lactobacillus and g-Eubacterium_xylanophilum_group) and another in which other genera decreased in abundance (g-Tuzzerella, g-Roseburia, g-Lachnoclostridium or g-Escherichia_Shigella). When we focused only on the **TG** mice fed the three diets, a LEfSe analysis showed significant differences in 42 genera, of which 26 were common to all the groups compared (**TG, WT, MC, M**), whereas some were modified by the **H** diet, such as g-Prevotellaceae_UCG001, g-Lachnoclostridium, g-Tuzzerella, g-Escherichia_Shigella or g-Lactobacillus.
The relative abundance of Firmicutes is significantly higher when a high-fat diet is maintained (Jo et al.,2021), as seen for Lactococcus, Blautia, Lachnoclostridium, Oscillibacter, Ruminiclostridium, Harryflintia, Lactobacillus, Oscillospira and Erysipelatoclostridium. Our comparative LEfSe analysis showed some of these genera to be modified in **TH** and **THC** mice, such as g-Colidextribacter, g-Incertae-Sedis or g-Harryflintia, even though this diet is a low ω-3 diet and not really a high fat diet. Elsewhere, a higher abundance of pro-inflammatory Escherichia/Shigella bacteria has been reported in cognitively-impaired older adults without an AD diagnosis (Zhuang et al. 2020). Similarly, an increase in g-Escherichia/Shigella and a reduction of g-Ruminococcaceae, was proposed to be associated with a peripheral inflammatory status of AD patients with cognitive impairment and brain amyloidosis (Cattaneo et al. 2017). Interestingly, data from both genera correlated with our data in mice maintained on a **H** diet. Moreover, a decrease in the abundance of Lactobacillus *reuter*i in humans has been correlated with an increase in the incidence of inflammatory diseases (Mu et al. 2018), and L. *reuteri* induces an increase in gut intraepithelial $CD4^{+}CD8\alpha\alpha^{+}$ T cells (Cervantes-Barragan et al. 2017)**.** Thus, the decrease in g-Lactobacillus when mice follow a H diet strongly suggests a correlation between dysbiosis and a pathology.

Some controversial data exists regarding g-Blautia, and while our **S** and **H** diets strongly increased g-Blautia, low levels of Blautia have been described in correlation with some pathologies. For instance, g-Blautia was negatively correlated with visceral fat accumulation in some human populations, and this genus has the ability to produce bactericins, among other compounds. It was reported that oral administration of Blautia wexlerae to mice induces metabolic changes and anti-inflammatory effects that decrease both high-fat diet–induced obesity and diabetes (Jo et al.,2021), whereas two Blautia species have been associated with visceral fat accumulation (Ozato et al. 2022) .

In summary our data show that the initial mice colony did not present any major differences in GM when maintained on our standard diet, as witnessed when the alpha or beta diversity between TG and WT mice or M and F mice was compared. However, the loss of sex hormones in males generated an important shift in GM diversity, with some genera approximating to that seen in females. A more detailed analysis of these

three groups showed the increase/decrease in several genera and thus, we conclude that these GM adjustments are likely to be associated with sex hormone levels. Accordingly, the levels of sex hormones are something to take into account when studying the impact of diets on human GM populations.

We also analysed the effect of three diets with different ratios of ω-3/ω-6 FAs, and the diet with less ω-3 FAs generated significant changes in GM diversity, affecting more the 40 genera. In addition, we observed some differences between WT and TG mice that followed these diets that suggest additional genetic factors (APP and PS1 mutations in our mouse model) can influence GM diversity. We observed some shifts in genera (increases/decreases) associated with the **H** diet. Considering all the data, it is tantalizing to propose that a combination (reduction/increase) of these genera may be responsible for the intensity of the neuropathology in APP/PS1 mice fed a **H** diet, mostly due to the low levels of ω-3-FAs. These data strongly suggest that a reorganisation of the GM and not the modification of one specific genus should be considered as responsible for the deterioration of the AD neuropathology in our mouse model.

Acknowledgments

This work was supported by grants from the Spanish FEDER/Science and Ministry for Innovation I+D+i-RETOS-PID2021-124801NB-I00, and from the Centro de Investigación Biomédica en Red sobre Enfermedades Neurodegenerativas (CIBERNED; an initiative of the ISCIII - [PI2016/01]). Institutional grants from the Fundación Ramón Areces and Banco Santander to the Centro de Biología Molecular Severo Ochoa (CBMSO: CSIC-UAM, Madrid, Spain) are also acknowledged by FW (CBMSO). The data analysis and submission to a public repository has been performed by the Biocomputational Analysis Core Facility (https://www.cbm.uam.es/index.php/facilities/biocomputational-analysis/) at the Centro de Biología Molecular (CBM) Severo Ochoa (CSIC-UAM) Madrid, Spain, which obtains general funding from both institutions. The Biocomputational Analysis facility also receives funding from the European Commission – NextGenerationEU (Regulation EU 2020/2094), through CSIC's Global Health Platform (PTI+ Salud Global)."

We especially want to thank **M**aria **S**antos **G**alindo. for the help, teaching and advice during the analysis process with the program MicrobiomeAnalyst 2.0.

The manuscript was edited and revised by Dr. **M**arck **S**efton from **BiomedRed**.

Author Contributions

Conceptualization, FW, ; Methodology, LO-G; Data Curation, FW supported by Biocomputational Facility -MSG), ; Writing—Original Draft, FW ; Writing—Review and Editing, FW, LO-G (Edited by BiomedRed-MS) ; Supervision, FW; Funding Acquisition, FW.

Disclosure statements

The authors declare that they have no competing interests. The funding bodies played no role in the study design, data collection and analysis, nor in the decision to publish or in the preparation of the manuscript.

FIGURE LEGENDS

FIGURE 1

A-Schematic representation of the experiments with different genotypes and diets. We fed APP/PS1 double transgenic mice and wildtype littermates; castrated (**MC**) and non-castrated (**M**) with three different diets (**S, H** and **L**), containing different ω–6/ ω–3 ratio. In addition, a group of APP/PS1 and WT females fed only with **S** diet was used as control having the full repertoire of sexual hormones.

B- Plots show the alpha analysis from the three sexual groups considered (F, M and MC), using Shannon test. The below data indicate the Kluskal-Wallis statistical analysis in which we represented p and DFR values.

C-The stacked bar plots represent the relative abundance profile in F, M and MC mice groups, at genus taxonomic rank. Some obvious differences were marked with colour arrows.

FIGURE 2

A- Plots show beta diversity analysis from sexual mice groups considered (F, M and MC), using Bray Curtis, at genus and feature-level. The corresponding statistical analysis was indicated below, representing p and DFR values.

B-The data from sexual mice groups considered (F, M and MC), were additionally analysed using Linear Discrimination Test (LEfSe) showing two phyla significantly different, one in F group and one in MC group.

C-Plots show Linear Discrimination Analysis (LDA) from F, M and MC mice. Data shows some genera represented when considering LDA >2.

FIGURE 3

A-Plots show the alpha analysis from the three sexual groups and the two genotypes considered (FW, FT, WS. WSC and TS, TSC), using Shannon test. The asterisks (**- $p<0.001$) represent p value of the Kluskal-Wallis statistical analysis.

B- The stacked bar plots represent the relative abundance profile in FW, FT, WS. WSC and TS, TSC mice groups, at genus taxonomic rank. Some obvious differences were marked with colour arrows.

C- Plots show beta diversity analysis from FW, FT, WS. WSC and TS, TSC mice groups, using Bray Curtis, at feature-level. The corresponding statistical analysis was represented on the right table, representing p and DFR values. (Note that p values were represented in bold only when with FDR below 0.03.)

FIGURE 4

A-Plots show the alpha analysis at phylum and genus level, from the three diets used (S, L and H), using Shannon test. The table represent the represent p value and FDR value from Kluskal-Wallis statistical analysis. (Note that p values were represented in bold only when with FDR below 0.03.)

B- The stacked bar plots represent the relative abundance profile of microbiota after feeding with S, or L or H diets, at phylum taxonomic rank. Some obvious differences were marked with colour arrows.

C- The stacked bar plots represent the relative abundance profile of microbiota after feeding with S, or L or H diets, at genus taxonomic rank. The colour arrows match with the corresponding genera in the list, and some evident differences were obvious.

FIGURE 5

A-Plots show beta diversity analysis from mice groups feed with each diet (S, L and H), using Bray Curtis test, at genus taxonomic rank. The corresponding statistical analysis was represented on the right table, representing p and DFR values.

B. Plots show beta diversity analysis from mice groups feed with each diet (S, L and H), using Bray Curtis, at feature-level taxonomic rank.

In both cases (A, B) the corresponding statistical analysis was represented on the right table, representing p and DFR values. (Note that p values were represented in bold only when with FDR below 0.04.)

C- Plots show Linear Discrimination Analysis (LDA) from mice fed with S, L and H diets. Data at genus taxonomic rank, show some genera more abundant in each diet, represented when considering LDA >2.

FIGURE 6

A-Plots show beta diversity analysis from mice groups feed with each diet, when we additionally considered genotype and sexual groups. Considering genotypes, we represented WS, WH, WL; WSC, WHC, WLC (wild-type); and TS, TH, TL, TSC, THC, TLC (APP/PS1), using Bray Curtis test, at genus taxonomic rank.

B.-Plots show Linear Discrimination Analysis (LDA) from above mice groups, at genus taxonomic rank, show some genera more abundant in each diet/genotype, represented when considering LDA >2.

REFERENCES

Baidoon N., Sanger G-J. The human colon: Evidence for degenerative changes during aging and the physiological consequences. Neurogastroenterology & Motility. 2024;00:e14848.

Barberger-Gateau P. Fish, meat, and risk of dementia: cohort study. BMJ. 2002 Oct 26;325(7370):932–3.

Barron AM, Rosario ER, Elteriefi R, Pike CJ. Sex-Specific Effects of High Fat Diet on Indices of Metabolic Syndrome in 3xTg-AD Mice: Implications for Alzheimer's Disease. PLoS One. 2013 Oct 28;8(10): e78554.

Belkaid Y, Hand TW. Role of the microbiota in immunity and inflammation. Vol. 157, Cell. 2014.

Bellenguez C, Küçükali F, Jansen IE, Kleineidam L, Moreno-Grau S, Amin N, et al. New insights into the genetic etiology of Alzheimer's disease and related dementias. Nat Genet. 2022 Apr 4;54(4):412–36.

Burlingame B, Nishida C, Uauy R, Weisell R. Fats and Fatty Acids in Human Nutrition: Introduction. Ann Nutr Metab. 2009;55(1–3):5–7.

Calon F, Lim GP, Yang F, Morihara T, Teter B, Ubeda O, et al. Docosahexaenoic Acid Protects from Dendritic Pathology in an Alzheimer's Disease Mouse Model. Neuron. 2004 Sep;43(5):633–45.

Catalan J, Toru M, Slotnick B, Murthy M, Greiner RS, Salem N. Cognitive deficits in docosahexaenoic acid-deficient rats. Behavioral Neuroscience. 2002;116(6):1022–31.

Cattaneo A, Cattane N, Galluzzi S, Provasi S, Lopizzo N, Festari C, et al. Association of brain amyloidosis with pro-inflammatory gut bacterial taxa and peripheral inflammation markers in cognitively impaired elderly. Neurobiol Aging. 2017 Jan; 49:60–8.

Cederholm T, Palmblad J. Are omega-3 fatty acids options for prevention and treatment of cognitive decline and dementia? Curr Opin Clin Nutr Metab Care. 2010 Mar;13(2):150–5.

Cervantes-Barragan L, Chai JN, Tianero MaD, Di Luccia B, Ahern PP, Merriman J, et al. *Lactobacillus reuteri* induces gut intraepithelial $CD4^+$ $CD8\alpha\alpha^+$ T cells. Science (1979). 2017 Aug 25;357(6353):806–10.

Crawford MA, Broadhurst CL. The role of docosahexaenoic and the marine food web as determinants of evolution and hominid brain development: The challenge for human sustainability. Nutr Health. 2012 Jan 1;21(1):17–39.

Ettcheto M, Petrov D, Pedrós I, de Lemos L, Pallàs M, Alegret M, et al. Hypercholesterolemia and neurodegeneration. Comparison of hippocampal phenotypes in LDLr knockout and APPswe/PS1dE9 mice. Exp Gerontol. 2015 May; 65:69–78.

Feng M, Hou T, Zhou M, Cen Q, Yi T, Bai J, et al. Gut microbiota may be involved in Alzheimer's disease pathology by dysregulating pyrimidine metabolism in APP/PS1 mice. Front Aging Neurosci. 2022 Aug 3;14.

Fishbein SRS, Mahmud B, Dantas G. Antibiotic perturbations to the gut microbiome. Nat Rev Microbiol. 2023 Dec 25;21(12):772–88.

Frost G, Sleeth ML, Sahuri-Arisoylu M, Lizarbe B, Cerdan S, Brody L, et al. The short-chain fatty acid acetate reduces appetite via a central homeostatic mechanism. Nat Commun. 2014 Apr 29;5(1):3611.

Gandy K., Zhang J, Nagarkatti P, Nagarkatti M. The role of gut microbiota in shaping the relapse-remitting and chronic-progressive forms of multiple sclerosis in mouse models. Sci Rep. 2019 May 6;9(1):6923.

Green KN, Martinez-Coria H, Khashwji H, Hall EB, Yurko-Mauro KA, Ellis L, et al. Dietary Docosahexaenoic Acid and Docosapentaenoic Acid Ameliorate Amyloid-β and Tau Pathology via a Mechanism Involving Presenilin 1 Levels. The Journal of Neuroscience. 2007 Apr 18;27(16):4385–95.

Hardy J, Selkoe DJ. The Amyloid Hypothesis of Alzheimer's Disease: Progress and Problems on the Road to Therapeutics. Science (1979). 2002 Jul 19;297(5580):353–6.

Herrera JL, Ordoñez-Gutierrez L, Fabrias G, Casas J, Morales A, Hernandez G, et al. Ovarian Function Modulates the Effects of Long-Chain Polyunsaturated Fatty Acids on the Mouse Cerebral Cortex. Front Cell Neurosci. 2018 Apr 24;12.

Herrera JL, Ordoñez-Gutierrez L, Fabrias G, Casas J, Morales A, Hernandez G, et al. Ovarian Hormone-Dependent Effects of Dietary Lipids on APP/PS1 Mouse Brain. Front Aging Neurosci. 2019;11.

Hooijmans CR, Rutters F, Dederen PJ, Gambarota G, Veltien A, van Groen T, et al. Changes in cerebral blood volume and amyloid pathology in aged Alzheimer APP/PS1 mice on a docosahexaenoic acid (DHA) diet or cholesterol enriched Typical Western Diet (TWD). Neurobiol Dis. 2007 Oct;28(1):16–29.

Hooper L V., Littman DR, Macpherson AJ. Interactions between the microbiota and the immune system. Vol. 336, Science. 2012.

Huang WJ, Zhaang X, Chen WW. Role of oxidative stress in Alzheimer's disease. Biomed Rep. 2016 May;4(5):519–22.

Ikemoto A, Ohishi M, Sato Y, Hata N, Misawa Y, Fujii Y, et al. Reversibility of n-3 fatty acid deficiency-induced alterations of learning behavior in the rat: level of n-6 fatty acids as another critical factor. J Lipid Res. 2001 Oct;42(10):1655–63.

Ivanov II, Honda K. Intestinal Commensal Microbes as Immune Modulators. Cell Host Microbe. 2012 Oct;12(4):496–508.

Jankowsky JL, Slunt HH, Ratovitski T, Jenkins NA, Copeland NG, Borchelt DR. Co-expression of multiple transgenes in mouse CNS: a comparison of strategies. Biomol Eng. 2001 Jun;17(6):157–65.

Jo J., Seo, S., Park,S. , Kim H., Kim E., Kim J., et al. Gut Microbiome and Metabolome Profiles Associated with High-Fat Diet in Mice. Metabolites, 2021 Jul 27;11(8):482. doi: 10.3390/metabo11080482.

Levy-Lahad E, Wasco W, Poorkaj P, Romano DM, Oshima J, Pettingell WH, et al. Candidate Gene for the Chromosome 1 Familial Alzheimer's Disease Locus. Science 1979. 1995 Aug 18;269(5226):973–7.

Liu P, Wu L, Peng G, Han Y, Tang R, Ge J, et al. Altered microbiomes distinguish Alzheimer's disease from amnestic mild cognitive impairment and health in a Chinese cohort. Brain Behav Immun. 2019 Aug; 80:633–43.

Man AL, Gicheva N, Nicoletti C. The impact of ageing on the intestinal epithelial barrier and immune system. Cell Immunol.2014;289:112-118. doi: 10.1016/j.cellimm.2014.04.001

Minter MR, Zhang C, Leone V, Ringus DL, Zhang X, Oyler-Castrillo P, et al. Antibiotic-induced perturbations in gut microbial diversity influences neuro-inflammation and amyloidosis in a murine model of Alzheimer's disease. Sci Rep. 2016 Jul 21;6(1):30028.

Morris MC, Evans DA, Bienias JL, Tangney CC, Bennett DA, Wilson RS, et al. Consumption of Fish and n-3 Fatty Acids and Risk of Incident Alzheimer Disease. Arch Neurol. 2003 Jul 1;60(7):940.

Mu Q, Tavella VJ, Luo XM. Role of Lactobacillus reuteri in Human Health and Diseases. Front Microbiol. 2018 Apr 19;9.

Mullan M, Crawford F, Axelman K, Houlden H, Lilius L, Winblad B, et al. A pathogenic mutation for probable Alzheimer's disease in the APP gene at the N–terminus of β–amyloid. Nat Genet. 1992 Aug;1(5):345–7.

Munir MU, Ali SA, Chung KHK, Kakinen A, Javed I, Davis TP. Reverse engineering the Gut-Brain Axis and microbiome-metabolomics for symbiotic/pathogenic balance in neurodegenerative diseases. Gut Microbes. 2024 Dec 31;16(1).

Ordóñez-Gutiérrez L, Antón M, Wandosell F. Peripheral amyloid levels present gender differences associated with aging in AβPP/PS1 mice. Journal of Alzheimer's Disease. 2015;44(4).

Ordóñez-Gutiérrez L, Fábrias G, Casas J, Wandosell F. Diets with Higher ω-6/ω-3 Ratios Show Differences in Ceramides and Fatty Acid Levels Accompanied by Increased Amyloid-Beta in the Brains of Male APP/PS1 Transgenic Mice. Int J Mol Sci. 2021 Oct 9;22(20):10907.

O'Toole PW, Jeffery IB. Gut microbiota and aging. Vol. 350, Science. 2015.

Ozato N, Yamaguchi T, Mori K, Katashima M, Kumagai M, Murashita K, et al. Two Blautia Species Associated with Visceral Fat Accumulation: A One-Year Longitudinal Study. Biology (Basel). 2022 Feb 16;11(2):318.

Park SH, Kim JH, Choi KH, Jang YJ, Bae, SS, Choi, BT, et al. Hypercholesterolemia accelerates amyloid β-induced cognitive deficits. Int J Mol Med. 2013 Mar;31(3):577–82.

Park,J.Y., Choi,J., Lee, Y., Lee, J.E., Lee, E.H., Kwon, H.J. et al.Metagenome Analysis of Bodily Microbiota in a Mouse Modelof Alzheimer Disease Using Bacteria-derived Membrane Vesicles in Blood,Exp Neurobiol 2017, 26 : 369–379.

Parker BJ, Wearsch PA, Veloo ACM, Rodriguez-Palacios A. The Genus Alistipes: Gut Bacteria with Emerging Implications to Inflammation, Cancer, and Mental Health. Front Immunol. 2020 Jun 9;11.

Pellegrini C, Fornai M, D'Antongiovanni V,Antonioli L, Bernardini N, Derkinderen P. Theintestinal barrier in disorders of the central nervous system. Lancet Gastroenterol Hepatol. 2023; 8:66–80.

Perez SE, Berg BM, Moore KA, He B, Counts SE, Fritz JJ, et al. DHA diet reduces AD pathology in young APPswe/PS1ΔE9 transgenic mice: Possible gender effects. J Neurosci Res. 2010 Apr 26;88(5):1026–40.

Ricciarelli R, Canepa E, Marengo B, Marinari UM, Poli G, Pronzato MA, et al. Cholesterol and Alzheimer's disease: A still poorly understood correlation. IUBMB Life. 2012 Dec 2;64(12):931–5.

Rosser EC, de Gruijter NM, Matei DE. Mini-Review: Gut-Microbiota and the Sex-Bias in Autoimmunity – Lessons Learnt from Animal Models. Front Med (Lausanne). 2022 Jun 16;9.

Sambra V, Echeverria F, Valenzuela A, Chouinard-Watkins R, Valenzuela R. Docosahexaenoic and Arachidonic Acids as Neuroprotective Nutrients throughout the Life Cycle. Nutrients. 2021 Mar 18;13(3):986.

Scarmeas N, Stern Y, Tang M, Mayeux R, Luchsinger JA. Mediterranean diet and risk for Alzheimer's disease. Ann Neurol. 2006 Jun 18;59(6):912–21.

Selkoe DJ. Alzheimer's Disease Is a Synaptic Failure. Science (1979). 2002 Oct 25;298(5594):789–91.

Sheng C, Yang K, He B, Du W, Cai Y, Han Y. Combination of gut microbiota and plasma amyloid-β as a potential index for identifying preclinical Alzheimer's disease: a cross-sectional analysis from the SILCODE study. Alzheimers Res Ther. 2022 Dec 14;14(1):35.

Sherrington R. Alzheimer's disease associated with mutations in presenilin 2 is rare and variably penetrant. Hum Mol Genet. 1996 Jul 1;5(7):985–8.

Silva YP, Bernardi A, Frozza RL. The Role of Short-Chain Fatty Acids from Gut Microbiota in Gut-Brain Communication. Front Endocrinol (Lausanne). 2020 Jan 31;11.

Sophocleous A, Idris AI. Ovariectomy/Orchiectomy in Rodents. In 2019. p. 261–7.

Spadoni I, Zagato E, Bertocchi A, Paolinelli R, Hot E, Di Sabatino A, et al. A gut-vascular barrier controls the systemic dissemination of bacteria. Science (1979). 2015 Nov 13;350(6262):830–4.

Verhaar BJH, Hendriksen HMA, de Leeuw FA, Doorduijn AS, van Leeuwenstijn M, Teunissen CE, et al. Gut Microbiota Composition Is Related to AD Pathology. Front Immunol. 2022 Jan 31;12.

Vernocchi P, Del Chierico F, Putignani L. Gut Microbiota Profiling: Metabolomics Based Approach to Unravel Compounds Affecting Human Health. Front Microbiol. 2016 Jul 26;7.

Vogt NM, Kerby RL, Dill-McFarland KA, Harding SJ, Merluzzi AP, Johnson SC, et al. Gut microbiome alterations in Alzheimer's disease. Sci Rep. 2017 Oct 19;7(1):13537.

Wang, Y., Huang, JM., Zhou M., Cen Q., Yi, T. Bai J., et al., Gut microbiota may be altered in Alzheimer disease pathology by dysregulation pyrimidine metabolism in APP/PS1 mice. Front. Aging Neurosci. 2022, Aug3., 14.

Zhang L, Wang Y, Xiayu X, Shi C, Chen W, Song N, et al. Altered Gut Microbiota in a Mouse Model of Alzheimer's Disease. Journal of Alzheimer's Disease. 2017 Nov 7;60(4):1241–57.

Zhang, T., Gao, G., Kwok LY., et al. Gut microbiome-targeted therapies for Alzheimer's disease, Gut Microbes. 2023, Dec. 15:2, 2271613, DOI: 10.1080/19490976.2023.2271613

Zhou A, Yuan Y, Yang M, Huang Y, Li X, Li S, et al. Crosstalk Between the Gut Microbiota and Epithelial Cells Under Physiological and Infectious Conditions. Front Cell Infect Microbiol. 2022 Jan 27;12.

Zhuang Z, Yang R, Wang W, Qi L, Huang T. Associations between gut microbiota and Alzheimer's disease, major depressive disorder, and schizophrenia. J Neuroinflammation. 2020 Dec 2;17(1):288.

Zhuang ZQ, Shen LL, Li WW, Fu X, Zeng F, Gui L, et al. Gut Microbiota is Altered in Patients with Alzheimer's Disease. Journal of Alzheimer's Disease. 2018 May 30;63(4):1337–46.
