# Supplementary material for: Sex hormones and diets rich in polyunsaturated ω-6/ω-3 fatty acids modify microbiota distinctly in a mouse model of Alzheimer’s disease
Source: Gut Microbiome (Camb). 2025 Jun 18;6:e10. doi: 10.1017/gmb.2025.10005 (PMC12284841; doi:10.1017/gmb.2025.10005)
Supplement: Ordoñez-Gutierrez and Wandosell supplementary material [file S2632289725100054sup001.zip › FIGS- Figs Supl + Legends -11-03-2025 .pdf]

A

ALPHA DIVERSITY

Shanon-Kluskal-Wallis test PHYLUM

Pair	Statistic	P-value	FDR
TG vs WT	100.0	0.2039	0.2039

Shanon-Kluskal-Wallis test GENUS

Pair	Statistic	P-value	FDR
TG vs WT	133.0	0.92923	0.92923

B

BETA DIVERSITY-BRAY-CURTIS (Phylum)

Pair	F-value	R-squared	P-value	FDR
TG vs WT	0.16072	0.0051578	0.787	0.787

BETA DIVERSITY-BRAY-CURTIS (Genus)

Pair	F-value	R-squared	P-value	FDR
TG vs WT	0.82031	0.025779	0.494	0.494

C

Linear Discriminant Analysis Effect Size (LEfSe)

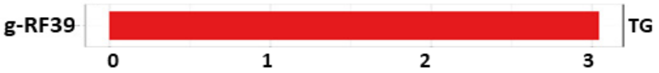

Name	Pvalues	FDR	TG	WT	LDAscore
g__RF39	**3.9641E-4**	0.020614	2210.8	0.0	3.04

D

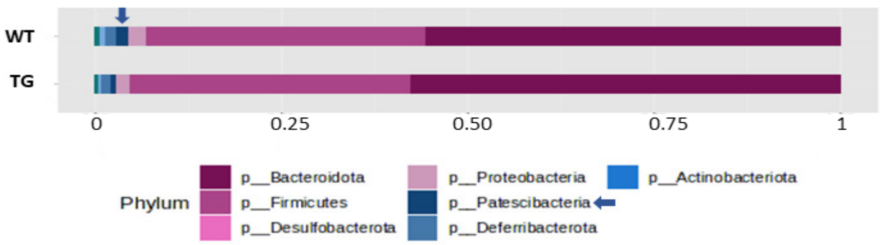

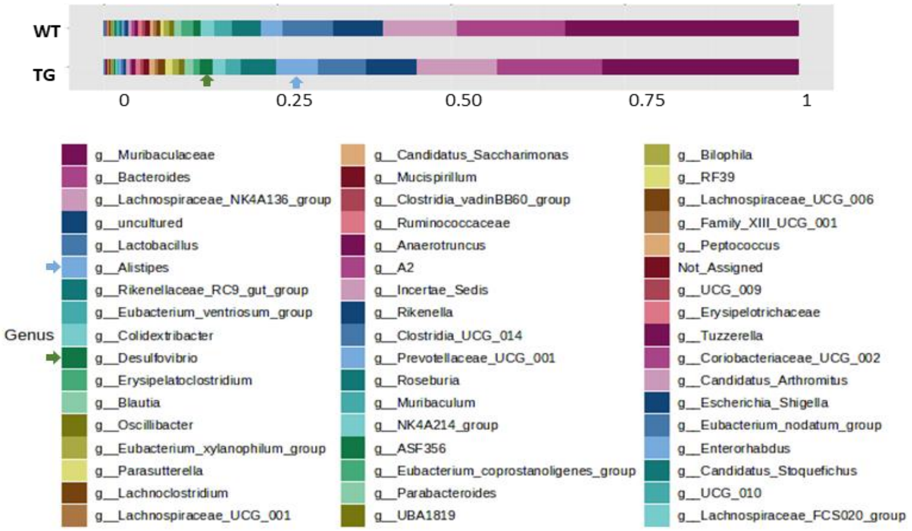

E

Single-factor Statistical Comparisons (Mann-Whitney/Kruskal-Wallis)

Name	log2FC	Pvalues	FDR	Statistics	logCPM
g__RF39	-10.4115	**4.3103E-4**	0.022413	216.0	9.70261

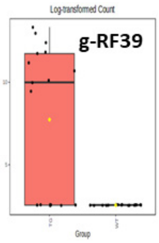

Supplementary Figure 1

A

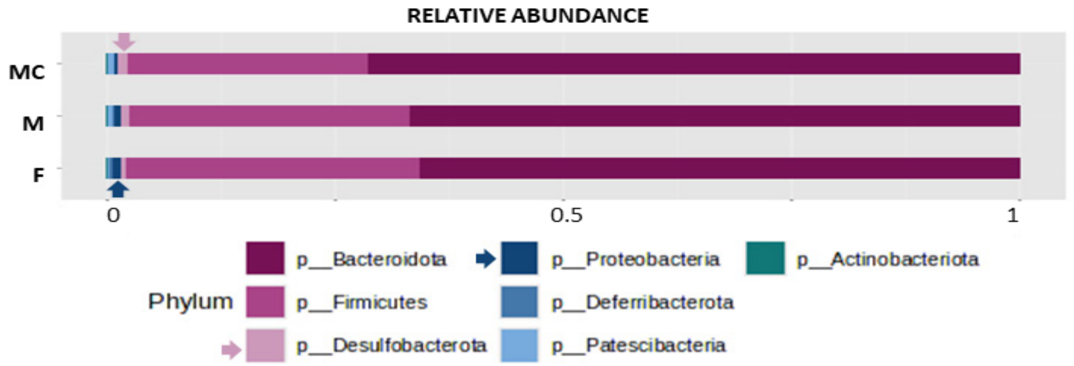

B

BETA DIVERSITY BRAY-CURTIS (Phylum)

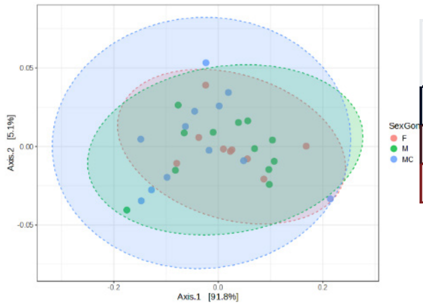

Pair	F-value	R-squared	P-value	FDR
**F vs MC**	2.1172	0.10026	0.152	0.3735
**F vs M**	0.23784	0.012363	0.704	0.704
**MC vs M**	1.4332	0.061161	0.249	0.3735

C

Linear Discriminant Analysis Effect Size (LEfSe)

Name	Pvalues	FDR	F	M	MC	LDAscore
**g__Eubacterium_ventriosum_group**	**9.9473E-5**	0.0050731	23612.0	3106.1	30288.0	4.13
**g__Lachnospiraceae_FCS020_group**	**2.7031E-4**	0.0054165	725.15	130.88	0.0	2.56
**g__Prevotellaceae_UCG_001**	**5.0945E-4**	0.0054165	1124.6	3158.5	1186.6	3.01
**g__Parasutterella**	**5.1475E-4**	0.0054165	7600.5	5098.4	823.07	3.53
**g__Alistipes**	**5.3103E-4**	0.0054165	25516.0	69056.0	19611.0	4.39
**g__Peptococcus**	**9.5509E-4**	0.0081183	178.38	1890.4	442.07	2.93
**g__Lactobacillus**	**0.0020136**	0.014671	81077.0	15278.0	35380.0	4.52
**g__Bacteroides**	**0.0030795**	0.019279	194590.0	58513.0	97026.0	4.83
**g__Rikenella**	**0.0036983**	0.019279	744.54	3318.4	3603.5	3.16
**g__Candidatus_Saccharimonas**	**0.0038701**	0.019279	674.74	4496.3	5214.7	3.36
**g__ASF356**	**0.0041583**	0.019279	3342.7	657.29	540.96	3.15
**g__uncultured**	**0.0045474**	0.019327	55856.0	81591.0	40929.0	4.31
**g__UCG_009**	**0.0049525**	0.019429	473.09	1268.0	279.2	2.69
**g__Colidextribacter**	**0.0056335**	0.020522	7329.1	15659.0	11508.0	3.62
**g__Eubacterium_nodatum_group**	**0.0064798**	0.022031	391.66	994.66	197.77	2.6
**g__Oscillibacter**	**0.0097924**	0.031213	3311.7	7212.7	3405.7	3.29
**g__Blautia**	**0.01271**	0.038131	1931.2	9597.6	2352.9	3.58
**g__Clostridia_UCG_014**	0.021093	0.059763	50.412	3801.2	1695.6	3.27
**Not_Assigned**	0.025938	0.069623	1531.7	2533.2	590.4	2.99
**g__Muribaculaceae**	0.030287	0.077232	397840.0	506120.0	554100.0	4.89

Supplementary Figure 2

Linear Discriminant Analysis Effect Size (LEfSe)

Name	Pvalues	FDR	F	M	MC	LDAscore
s__uncultured_bacterium	**0.0013222**	0.0092551	928930.0	953780.0	964730.0	4.25
Not_Assigned	**0.0054842**	0.017112	36921.0	19460.0	17936.0	3.98
s__Bacteroides_caecimuris	**0.0073338**	0.017112	18590.0	8445.9	5555.0	3.81
s__Lactobacillus_reuteri	0.05312	0.09296	4610.7	508.96	3306.8	3.31
s__uncultured_Bacteroidales	0.070293	0.098411	9380.4	15121.0	7634.5	3.57

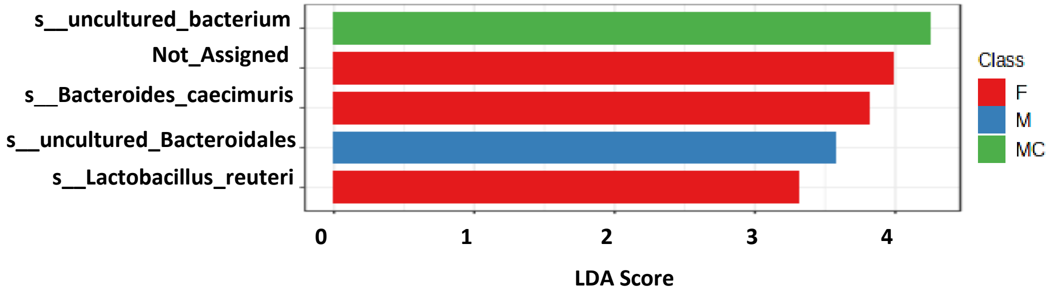

s__Bacteroides_caecimuris

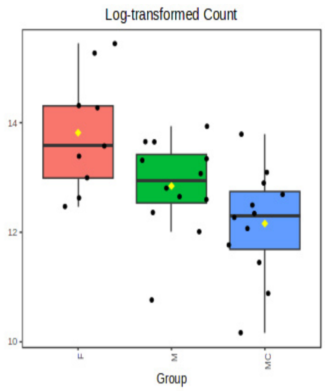

s__Lactobacillus_reuteri

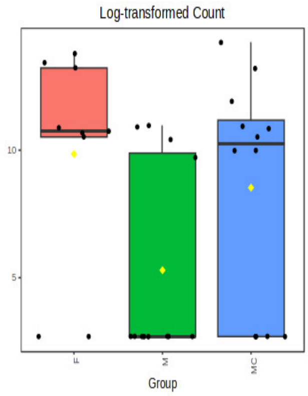

Supplementary Figure 3

ALPHA DIVERSITY - GROUPS

A

Shanon-Kluskal-Wallis
GENUS

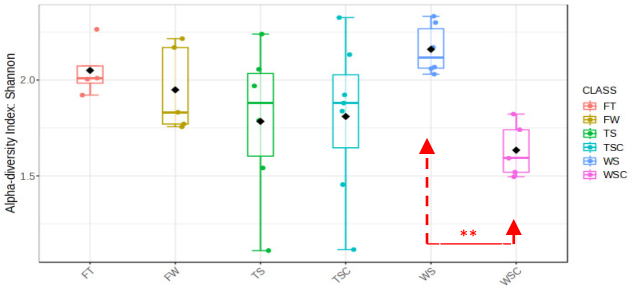

Shanon-Kluskal-Wallis
SPECIES

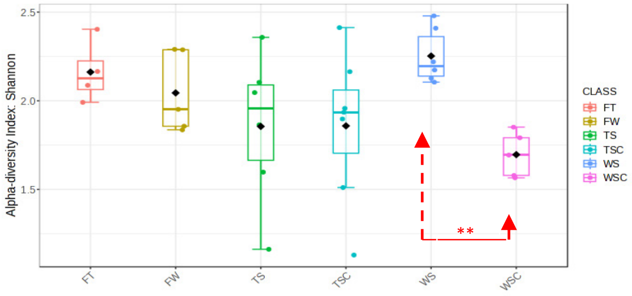

Pair	Statistic	P-value	FDR
**WSC vs WS**	0.0	**0.004329**	0.064935
**FT vs WSC**	20.0	0.015873	0.11905
**WS vs TS**	31.0	0.041126	0.16667
**TSC vs WS**	7.0	0.051282	0.16667
**FW vs WSC**	22.0	0.055556	0.16667
**FT vs WS**	4.0	0.11429	0.28571
**FT vs TSC**	21.0	0.2303	0.40152
**FW vs WS**	8.0	0.24675	0.40152
**WSC vs TS**	8.0	0.24675	0.40152
**WSC vs TSC**	10.0	0.26768	0.40152
**FT vs FW**	14.0	0.4127	0.56277
**FT vs TS**	16.0	0.47619	0.59524
**FW vs TS**	17.0	0.79221	0.91409
**TSC vs TS**	21.0	1.0	1.0
**FW vs TSC**	18.0	1.0	1.0

Pair	Statistic	P-value	FDR
**WSC vs WS**	0.0	**0.004329**	0.064935
**FT vs WSC**	20.0	0.015873	0.11905
**FW vs WSC**	23.0	0.031746	0.13112
**TSC vs WS**	6.0	0.034965	0.13112
**WS vs TS**	30.0	0.064935	0.19481
**FT vs TSC**	23.0	0.10909	0.27273
**FW vs WS**	8.0	0.24675	0.40152
**WSC vs TS**	8.0	0.24675	0.40152
**FT vs TS**	18.0	0.25714	0.40152
**WSC vs TSC**	10.0	0.26768	0.40152
**FT vs WS**	7.0	0.35238	0.48052
**FT vs FW**	14.0	0.4127	0.51587
**FW vs TSC**	21.0	0.63889	0.73718
**FW vs TS**	17.0	0.79221	0.84879
**TSC vs TS**	20.0	0.94522	0.94522

B

RELATIVE ABUNDANCE

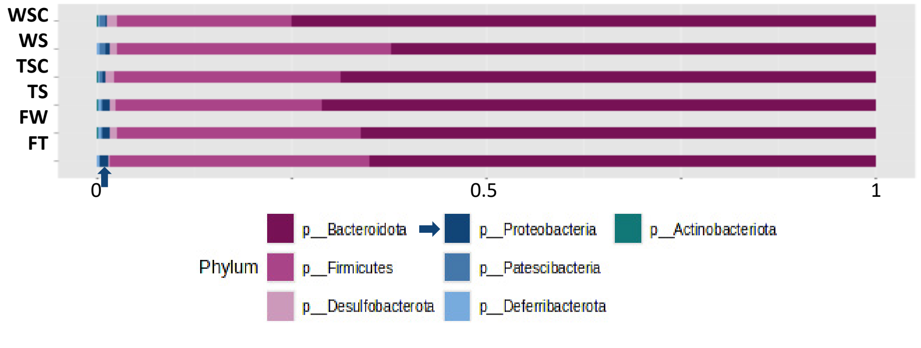

Supplementary Figure 4

A

BETA DIVERSITY-BRAY-CURTIS
(Genus Level)

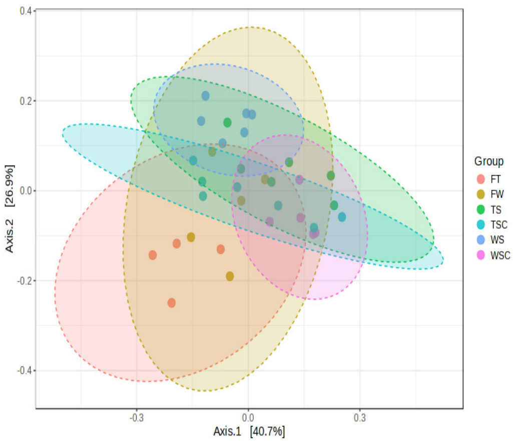

Pair	F-value	R-squared	P-value	FDR
**TSC vs WS**	4.149	0.27388	**0.003**	**0.015**
**FW vs WS**	4.7624	0.34605	**0.005**	**0.015**
**FT vs WSC**	11.638	0.62442	**0.006**	**0.015**
**FT vs WS**	12.856	0.61642	**0.006**	**0.015**
**FT vs TS**	9.1059	0.53232	**0.006**	**0.015**
**WSC vs WS**	15.373	0.63073	**0.006**	**0.015**
**FW vs WSC**	4.2859	0.34885	**0.01**	**0.021429**
**FW vs TS**	3.0789	0.2549	**0.019**	**0.033333**
**FT vs TSC**	4.2111	0.31876	**0.02**	**0.033333**
**WS vs TS**	4.2566	0.29857	**0.025**	**0.0375**
**WSC vs TS**	2.263	0.20092	0.064	0.087273
**FT vs FW**	2.2545	0.24361	0.106	0.12808
**WSC vs TSC**	2.2566	0.18412	0.111	0.12808
**TSC vs TS**	1.6672	0.13162	0.138	0.14786
**FW vs TSC**	0.80278	0.074312	0.546	0.546

B

Linear Discriminant Analysis (LDA)

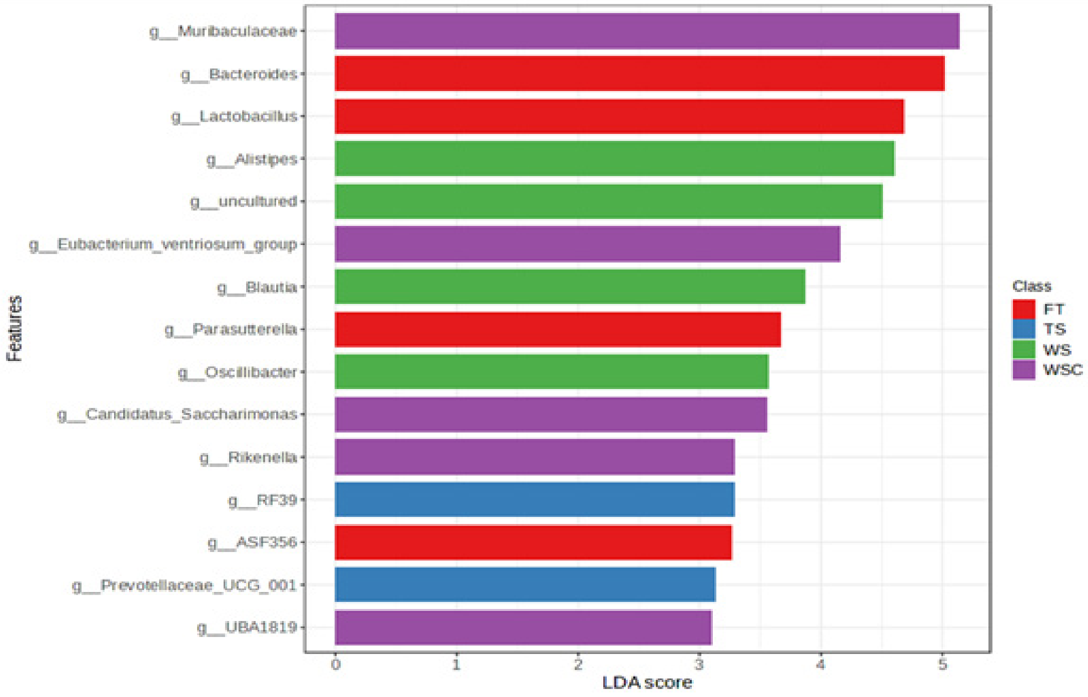

Supplementary Figure 5

Linear Discriminant Analysis Effect Size (LEfSe)

Phylum

Name	P-values	FDR	FT	FW	TS	TSC	WS	WSC
**p__Patescibacteria**	**0.0023135**	0.011406	462.43	844.59	2326.7	3564.8	6666.0	3.55
**p__Proteobacteria**	**0.0032588**	0.011406	9684.8	7573.4	6846.3	1710.1	4374.2	3.68

Genus

Name	P-values	FDR	FT	FW	TS	TSC	WS	WSC
**g__RF39**	**2.8838E-4**	0.014708	0.0	0.0	3821.6	1999.3	0.0	3.28
**g__Lachnospiraceae_FCS020_group**	**0.0018574**	0.019665	427.53	963.25	69.801	0.0	191.95	2.68
**g__Alistipes**	**0.0020243**	0.019665	21734.0	28542.0	42195.0	16952.0	95918.0	4.6
**g__Eubacterium_ventriosum_group**	**0.0020378**	0.019665	26891.0	20989.0	3612.2	29411.0	2600.1	4.16
**g__Peptococcus**	**0.0020876**	0.019665	104.7	237.32	1372.7	732.91	2408.1	3.07
**g__Candidatus_Saccharimonas**	**0.0023135**	0.019665	462.43	844.59	2326.7	3564.8	6666.0	3.55
**g__Parasutterella**	**0.0048378**	0.032568	9248.6	6282.1	6747.4	1301.3	3449.3	3.66
**g__Prevotellaceae_UCG_001**	**0.0051087**	0.032568	1378.6	921.37	3588.9	997.15	2728.0	3.13
**g__Muribaculaceae**	**0.0071891**	0.034466	333760.0	449100.0	581180.0	515150.0	431060.0	5.14
**g__UCG_009**	**0.0073794**	0.034466	732.91	265.24	1122.6	478.63	1413.5	2.85
**g__Blautia**	**0.0075002**	0.034466	1919.5	1940.5	2908.4	3041.3	16287.0	3.87
**g__NK4A214_group**	**0.0081095**	0.034466	261.75	1389.0	1931.2	299.15	1948.6	2.99
**g__uncultured**	**0.011283**	0.041897	57952.0	54179.0	73942.0	51383.0	89240.0	4.5
**g__Bacteroides**	**0.011814**	0.041897	257280.0	144450.0	53136.0	112730.0	63891.0	5.01
**g__Lactobacillus**	**0.012323**	0.041897	104050.0	62702.0	22016.0	21324.0	8539.0	4.68
**g__Oscillibacter**	**0.013898**	0.044299	2268.5	4146.2	5851.6	4886.1	8573.9	3.56
**g__ASF356**	0.01803	0.054089	3847.8	2938.6	738.72	802.71	575.86	3.26
**WSC**	0.023793	0.066208	0.0	1061.0	744.54	563.39	1128.4	3.1
**g__Rikenella**	0.024666	0.066208	1003.4	537.47	3466.8	3101.1	3170.1	3.28
**g__Eubacterium_nodatum_group**	0.027013	0.068884	689.28	153.56	785.26	279.2	1204.1	2.75

Supplementary Figure 6

Single-factor Statistical Comparisons (DESeq2)

WS/FW

Name	log2FC	lfcSE	P-values	FDR
g__Clostridia_UCG_014	-24.137	2.4239	**2.3276E-23**	1.1638E-21
g__Lactobacillus	3.5763	0.87531	**4.3925E-5**	0.0010981
g__Bacteroides	1.7581	0.57489	**0.0022268**	0.037114

g__Lactobacillus

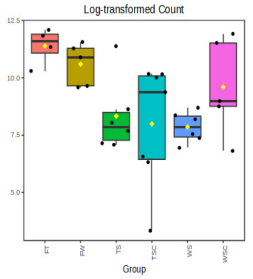

g__Clostridia_UCG014

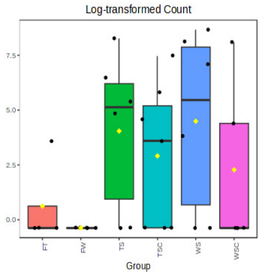

WS/TS

Name	log2FC	lfcSE	P-values	FDR
g__RF39	9.2224	2.0958	**1.0799E-5**	5.3997E-4

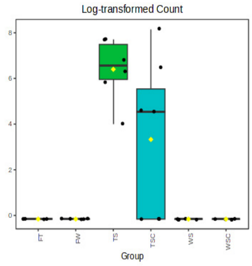

g__RF39

WS/WSC

Name	log2FC	lfcSE	P-values	FDR
g__Lachnospiraceae_UCG_001	-24.432	3.7926	**1.1784E-10**	5.8922E-9
g__UCG_009	-6.8705	1.5018	**4.7682E-6**	1.1921E-4
g__Lactobacillus	3.5178	0.87533	**5.8484E-5**	8.4505E-4
g__Peptococcus	-6.3845	1.6023	**6.7604E-5**	8.4505E-4
g__uncultured	-1.1266	0.33636	**8.0982E-4**	0.0067644
Not_Assigned	-7.0464	2.1042	**8.1173E-4**	0.0067644
g__Eubacterium_ventriosum_group	4.2241	1.4181	**0.0028956**	0.020683
g__Blautia	-2.6008	0.96637	**0.0071176**	0.037649
g__Alistipes	-1.2945	0.48297	**0.007356**	0.037649
g__Muribaculaceae	1.2287	0.45977	**0.0075299**	0.037649

g__Lachnospiraceae_UCG_001

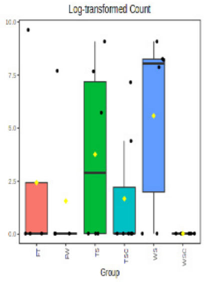

g-Eubacterium-ventriosum group

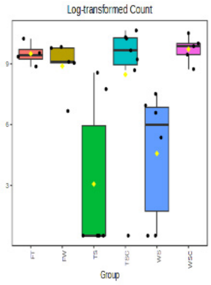

g-Peptococcus

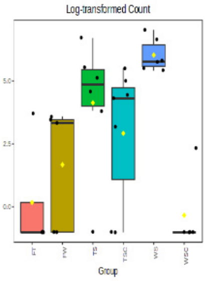

g-Alistipes

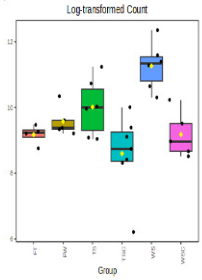

Supplementary Figure 7

A

Shanon-Kluskal-Wallis
PHYLUM

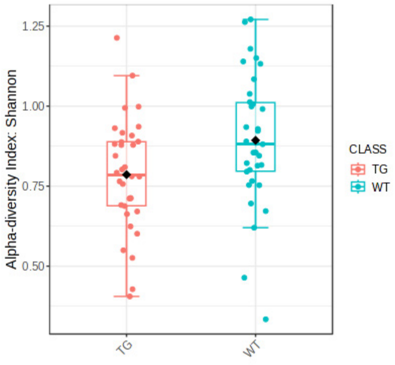

Pair	Statistic	P-value	FDR
TG vs WT	378.0	**0.013737**	0.013737

Shanon-Kluskal-Wallis
GENUS

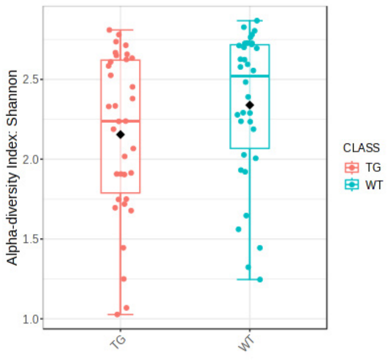

Pair	Statistic	P-value	FDR
TG vs WT	437.0	0.074198	0.074198

B

RELATIVE ABUNDANCE

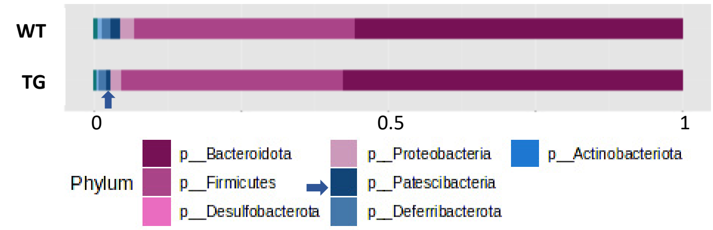

C

RELATIVE ABUNDANCE

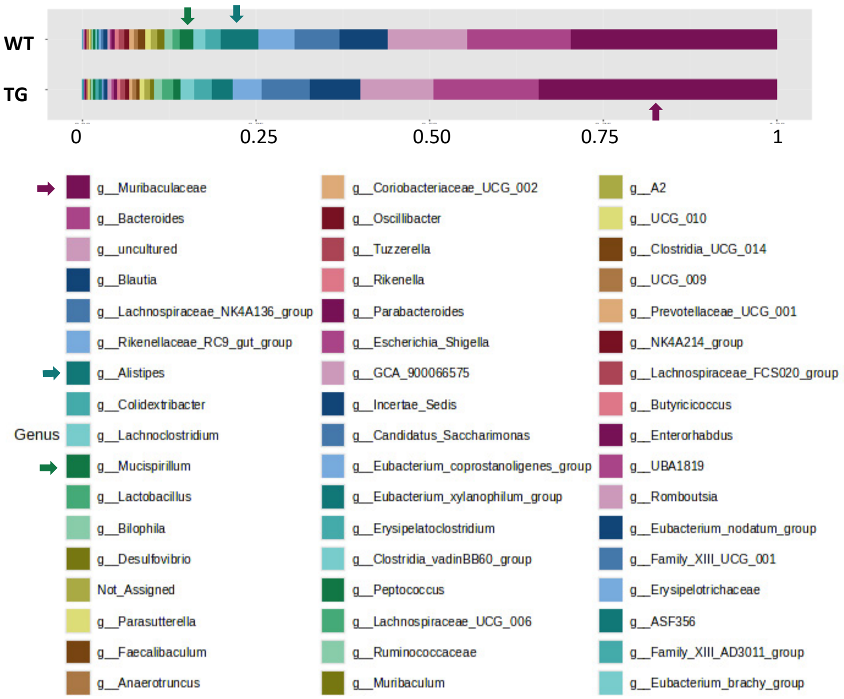

Supplementary Figure 8

A

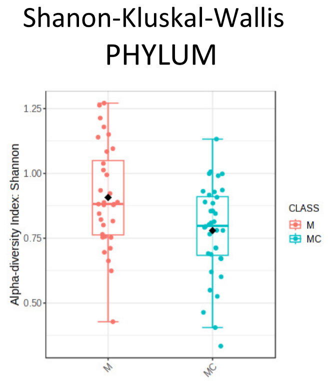

Pair	Statistic	P-value	FDR
**M vs MC**	779.0	**0.012184**	0.012184

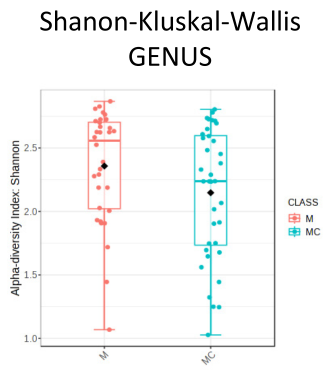

Pair	Statistic	P-value	FDR
**M vs MC**	742.0	0.04147	0.04147

B

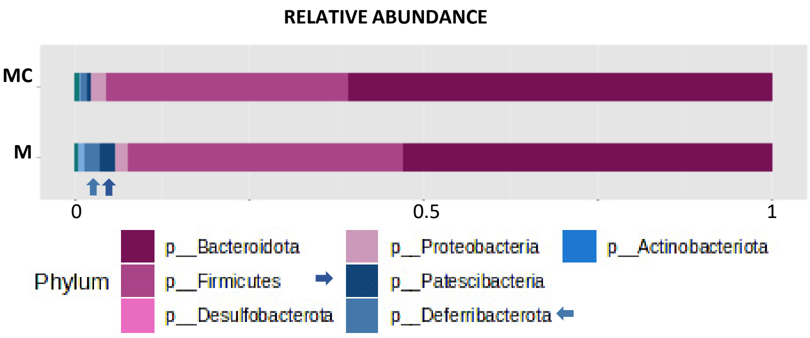

C

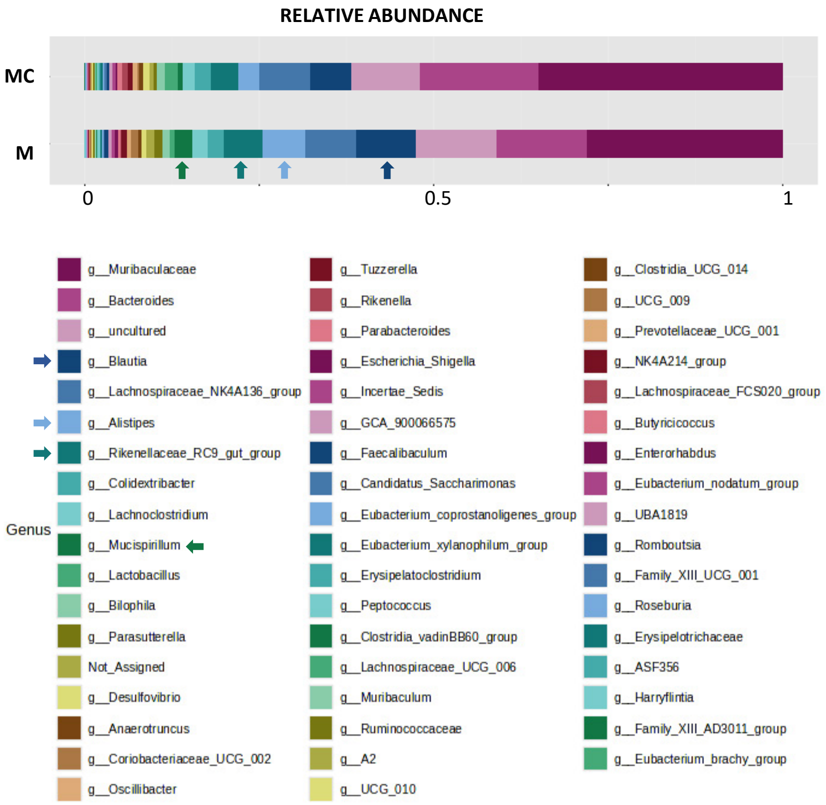

Supplementary Figure 9

BETA DIVERSITY BRAY-CURTIS

A

Pair	F-value	R-squared	P-value	FDR
TG .vs. WT	1.0368	0.015466	0.344	0.344

B

Genus level

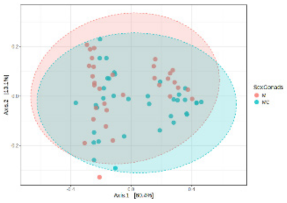

Pair	F-value	R-squared	P-value	FDR
M .vs. MC	2.6738	0.038935	0.057	0.057

Pair	F-value	R-squared	P-value	FDR
M .vs. MC	4.7969	0.067756	**0.004**	0.004

Feature level

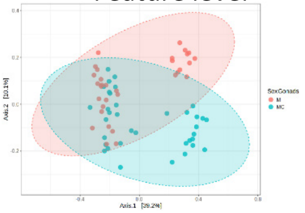

C

Linear Discriminant Analysis (LDA)

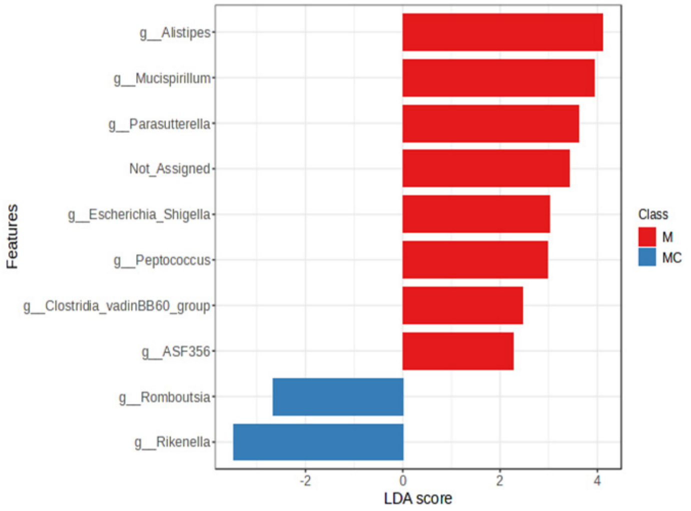

Linear Discriminant Analysis Effect Size (LEfSe)

Name	P-values	FDR	M	MC	LDAscore
**p__Deferribacterota**	**0.0016482**	0.0062726	23311.0	5726.6	3.94
**p__Proteobacteria**	**0.0017922**	0.0062726	19030.0	8816.2	3.71

Name	P-values	FDR	M	MC	LDAscore
**g__Rikenella**	**1.4462E-6**	7.5204E-5	1939.5	8019.7	-3.48
**g__Peptococcus**	**1.5248E-5**	3.9644E-4	2825.6	900.82	2.98
**g__Alistipes**	**4.3992E-5**	7.6252E-4	57206.0	31176.0	4.11
**g__Romboutsia**	**7.3245E-4**	0.0089357	113.57	1028.4	-2.66
**Not_Assigned**	**8.592E-4**	0.0089357	10444.0	5203.0	3.42
**g__Mucispirillum**	**0.0016482**	0.014285	23311.0	5726.6	3.94
**g__ASF356**	**0.0039815**	0.029577	595.32	224.09	2.27
**g__Clostridia_vadinBB60_group**	**0.0073736**	0.047929	1943.2	1372.3	2.46
**g__Escherichia_Shigella**	0.0091504	0.052869	5706.1	3614.4	3.02
**g__Parasutterella**	0.01262	0.065623	13324.0	5201.9	3.61

Supplementary Figure 10

Linear Discriminant Analysis Effect Size (LEfSe)

Name	P-values	FDR	H	L	S	LDAscore
**g__Prevotellaceae_UCG_001**	**1.7864E-11**	9.2895E-10	0.0	192.34	2265.0	3.05
**g__Bilophila**	**6.2674E-11**	1.6295E-9	22460.0	15992.0	872.17	4.03
**g__Tuzzerella**	**1.6502E-9**	1.9557E-8	10639.0	7687.0	658.27	3.7
**g__Blautia**	**1.8142E-9**	1.9557E-8	96303.0	117820.0	6718.7	4.74
**g__Muribaculaceae**	**1.8804E-9**	1.9557E-8	165920.0	204910.0	563890.0	5.3
**g__Lachnoclostridium**	**3.1937E-9**	2.7679E-8	29661.0	22849.0	4566.4	4.1
**g__Ruminococcaceae**	**4.6158E-9**	3.4289E-8	0.0	275.25	3593.1	3.25
**g__Lactobacillus**	**4.3383E-8**	2.8199E-7	797.88	11758.0	25779.0	4.1
**g__uncultured**	**8.2345E-8**	4.7577E-7	151070.0	121780.0	51265.0	4.7
**g__GCA_900066575**	**1.3962E-7**	7.2605E-7	7005.8	5689.0	606.87	3.51
**g__Butyricicoccus**	**1.6436E-7**	7.7696E-7	1603.7	744.49	76.273	2.88
**g__Eubacterium_xylanophilum_group**	**7.009E-7**	3.0372E-6	783.95	711.33	5025.7	3.33
**Not_Assigned**	**1.3458E-6**	5.3832E-6	12812.0	9370.0	1674.7	3.75
**g__Family_XIII_UCG_001**	**4.9388E-6**	1.8108E-5	47.754	197.32	1135.8	2.74
**g__Escherichia_Shigella**	**5.3925E-6**	1.8108E-5	9735.8	4591.3	397.95	3.67
**g__Lachnospiraceae_FCS020_group**	**5.5718E-6**	1.8108E-5	1251.5	1223.7	71.299	2.77
**g__Parabacteroides**	**1.2864E-5**	3.9349E-5	4998.2	8099.9	1200.5	3.54
**g__Mucispirillum**	**2.2433E-5**	6.4806E-5	15800.0	23267.0	3420.7	4.0
**g__Faecalibaculum**	**3.0877E-5**	8.4505E-5	4723.6	7239.3	164.15	3.55
**g__Erysipelatoclostridium**	**4.1613E-5**	1.0819E-4	0.0	3465.5	3041.0	3.24
**g__Incertae_Sedis**	**7.9447E-5**	1.9673E-4	6215.9	4259.7	2832.1	3.23
**g__Bacteroides**	**9.151E-5**	2.163E-4	182820.0	192260.0	84555.0	4.73
**g__Colidextribacter**	**1.5248E-4**	3.4474E-4	37586.0	25105.0	14838.0	4.06
**g__Desulfovibrio**	**3.8539E-4**	8.3501E-4	7998.7	3211.8	12154.0	3.65
**g__Anaerotruncus**	**5.5784E-4**	0.0011603	9556.7	7381.9	3669.4	3.47
**g__UBA1819**	**8.386E-4**	0.0016772	171.12	316.7	1416.0	2.79
**g__Coriobacteriaceae_UCG_002**	**0.0017691**	0.0034072	8496.2	10302.0	593.6	3.69
**g__Parasutterella**	**0.0022185**	0.0041201	7304.3	16014.0	3518.5	3.8
**g__Romboutsia**	**0.0026538**	0.0047585	1142.1	759.41	0.0	2.76
**g__A2**	**0.0035236**	0.0059157	286.52	582.0	2379.4	3.02
**g__Lachnospiraceae_UCG_006**	**0.0035267**	0.0059157	3796.4	379.71	1318.2	3.23
**g__Enterococcus**	**0.0045995**	0.0074742	525.29	114.41	49.743	2.38
**g__Roseburia**	**0.009483**	0.014943	961.04	523.96	0.0	2.68
**g__Muribaculum**	**0.013648**	0.020873	690.44	1653.1	1503.9	2.68
**g__Eubacterium_nodatum_group**	**0.017924**	0.02663	206.93	915.28	616.82	2.55
**g__NK4A214_group**	**0.019417**	0.028047	551.16	606.87	1369.6	2.61

Supplementary Figure 11

Single-factor Statistical Comparisons (DEReq2)

(FDR<0.04)

S vs H

Name	log2FC	lfcSE	P-values	FDR
g__Erysipelatoclostridium	-24.398	1.6009	**1.9212999999999995E-52**	9.7983999999999999E-51
g__Bilophila	3.7851	0.38321	**5.2186000000000004E-23**	1.3308E-21
g__Blautia	3.3518	0.3633	**2.8122999999999996E-20**	4.7809000000000005E-19
g__Muribaculaceae	-2.666	0.29107	**5.2310999999999995E-20**	6.6696E-19
g__Prevotellaceae_UCG_001	-8.8569	1.0936	**5.5494E-16**	5.6604E-15
g__Tuzzerella	3.4179	0.43656	**4.9102E-15**	4.1737E-14
g__Lactobacillus	-5.7487	0.78275	**2.0696E-13**	1.5079E-12
g__Ruminococcaceae	-9.6815	1.3398	**4.9653E-13**	3.1654E-12
g__Lachnoclostridium	2.2343	0.32992	**1.2682E-11**	7.1863E-11
g__Butyricicoccus	4.6415	0.74634	**5.001E-10**	2.3555E-9
g__Clostridia_UCG_014	-9.1181	1.4667	**5.0805E-10**	2.3555E-9
g__Escherichia_Shigella	4.3526	0.81859	**1.0539E-7**	4.4793E-7
g__uncultured	0.92617	0.1839	**4.7434E-7**	1.8609E-6
g__GCA_900066575	3.1715	0.68987	**4.2817E-6**	1.5597E-5
g__Lachnospiraceae_FCS020_group	3.8051	0.92064	**3.5783E-5**	1.2166E-4
g__Family_XIII_UCG_001	-5.1829	1.3149	**8.0908E-5**	2.5347E-4
g__Faecalibaculum	4.5939	1.1686	**8.4489E-5**	2.5347E-4
g__Romboutsia	6.3343	1.6374	**1.0951E-4**	3.1029E-4
Not_Assigned	2.211	0.61096	**2.958E-4**	7.94E-4
g__Lachnospiraceae_NK4A136_group	-0.91394	0.25566	**3.5048E-4**	8.9372E-4
g__UBA1819	-3.6582	1.0909	**7.988E-4**	0.0019399
g__Mucispirillum	1.9199	0.58289	**9.8878E-4**	0.0022922
g__Parabacteroides	1.4956	0.47007	**0.0014639**	0.0032461
g__Eubacterium_xylanophilum_group	-3.557	1.1329	**0.0016906**	0.0035925
g__Coriobacteriaceae_UCG_002	3.2381	1.0479	**0.002001**	0.004082
g__A2	-3.7078	1.4647	**0.01136**	0.022283
g__Candidatus_Saccharimonas	-1.532	0.61465	**0.012685**	0.02396
g__Muribaculum	-1.9525	0.82776	**0.018333**	0.033391
g__Erysipelotrichaceae	-2.3434	1.0212	**0.02174**	0.038232

L vs H

Name	log2FC	lfcSE	P-values	FDR
g__Erysipelatoclostridium	-24.486	1.5883	**1.2679999999999998E-53**	6.5934999999999984E-52
g__Lactobacillus	-4.3587	0.78467	**2.7801E-8**	7.2283E-7
g__Prevotellaceae_UCG_001	-5.2694	1.0953	**1.5035E-6**	2.6061E-5
g__Ruminococcaceae	-5.9152	1.3338	**9.2134E-6**	1.1977E-4

g-Lactobacillus

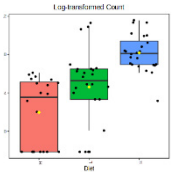

g-Tuzzerella

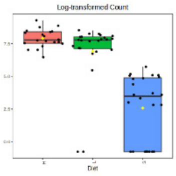

g-Roseburia

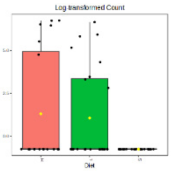

g-Lachnoclostridium

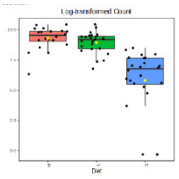

g-Escherichia_Shigella

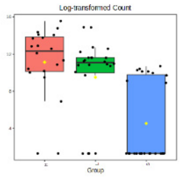

g-Eubacterium xylanophilum g.

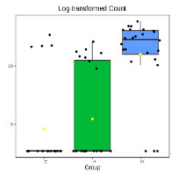

Supplementary Figure 12

Shanon-Kluskal-Wallis test PHYLUM

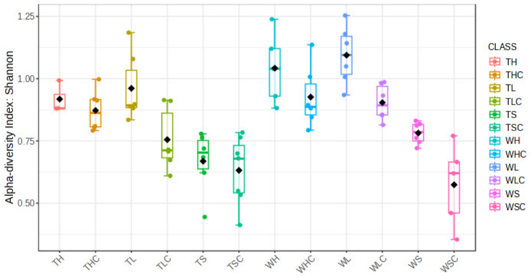

Pair	Statistic	P-value	FDR
**WLC vs TSC**	42.0	**0.0011655**	0.010989
**WLC vs TS**	36.0	**0.0021645**	0.010989
**THC vs TS**	36.0	**0.0021645**	0.010989
**WHC vs TS**	36.0	**0.0021645**	0.010989
**TL vs WS**	36.0	**0.0021645**	0.010989
**TL vs TS**	36.0	**0.0021645**	0.010989
**WL vs WS**	36.0	**0.0021645**	0.010989
**WL vs TS**	36.0	**0.0021645**	0.010989
**WLC vs WS**	34.0	**0.008658**	0.022857
**WHC vs WS**	33.0	**0.015152**	0.038462
**WLC vs TLC**	30.0	0.064935	0.11278
**WLC vs WSC**	30.0	**0.004329**	0.012987
**TLC vs TSC**	30.0	0.23427	0.32212
**WH vs WS**	30.0	**0.004329**	0.012987
**WH vs TS**	30.0	**0.004329**	0.012987
**WS vs TS**	30.0	0.064935	0.11278
**THC vs WS**	27.0	0.17965	0.25228
**WLC vs THC**	24.0	0.39394	0.48148
**TLC vs WSC**	24.0	0.12554	0.19805
**TLC vs TS**	21.0	0.69913	0.74424

Shanon-Kluskal-Wallis test GENUS

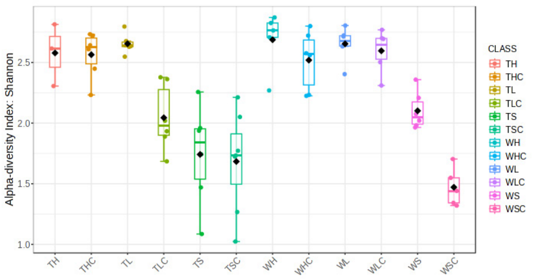

Pair	Statistic	P-value	FDR
**WLC vs TSC**	42.0	**0.0011655**	0.011905
**WLC vs TS**	36.0	**0.0021645**	0.011905
**TL vs WS**	36.0	**0.0021645**	0.011905
**TL vs TS**	36.0	**0.0021645**	0.011905
**WL vs WS**	36.0	**0.0021645**	0.011905
**WL vs TS**	36.0	**0.0021645**	0.011905
**WLC vs WS**	35.0	**0.004329**	0.012422
**THC vs WS**	35.0	**0.004329**	0.012422
**THC vs TS**	35.0	**0.004329**	0.012422
**WLC vs TLC**	34.0	**0.008658**	0.019048
**WHC vs WS**	34.0	**0.008658**	0.019048
**WHC vs TS**	34.0	**0.008658**	0.019048
**TLC vs TSC**	31.0	0.18065	0.27728
**WS vs TS**	31.0	**0.041126**	0.075397
**WLC vs WSC**	30.0	**0.004329**	0.012422
**WH vs TS**	30.0	**0.004329**	0.012422
**TLC vs WSC**	29.0	**0.008658**	0.019048
**WH vs WS**	29.0	**0.008658**	0.019048
**TLC vs TS**	25.0	0.30952	0.4256
**WLC vs WHC**	21.0	0.69913	0.78571

Supplementary Figure 13

BETA DIVERSITY GROUPS
GENUS

Pair	F-value	R-squared	P-value	FDR
**TSC vs THC**	15.019	0.57723	**0.001**	0.012375
**TSC vs WHC**	9.8025	0.47122	**0.001**	0.012375
**TSC vs TL**	20.31	0.64867	**0.001**	0.012375
**TL vs TS**	25.404	0.71755	**0.001**	0.012375
**WLC vs WSC**	36.68	0.80298	**0.002**	0.012375
**WSC vs TL**	31.694	0.77884	**0.002**	0.012375
**TSC vs WL**	15.327	0.58218	**0.002**	0.012375
**THC vs WS**	18.354	0.64731	**0.002**	0.012375
**WHC vs TS**	13.444	0.57346	**0.002**	0.012375
**WL vs WS**	18.781	0.65255	**0.002**	0.012375
**WLC vs TSC**	19.222	0.63603	**0.003**	0.012375
**WSC vs WL**	24.816	0.73385	**0.003**	0.012375
**WSC vs WS**	15.606	0.63424	**0.003**	0.012375
**TSC vs WS**	3.9571	0.26456	**0.003**	0.012375
**THC vs WH**	3.2835	0.26731	**0.003**	0.012375
**WH vs WS**	20.442	0.69432	**0.003**	0.012375
**TSC vs WH**	16.925	0.62859	**0.004**	0.013895
**THC vs TS**	20.0	0.66667	**0.004**	0.013895
**WL vs TS**	19.463	0.66059	**0.004**	0.013895
**WLC vs WS**	26.371	0.72506	**0.005**	0.014348

Supplementary Figure 14

Linear Discriminant Analysis Effect Size (LEfSe)

Name	P-values	FDR	TH	THC	TL	TLC	TS	TSC
**g__Prevotellaceae_UCG_001**	**3.8255E-8**	2.0275E-6	0.0	0.0	0.0	736.2	3734.1	3.27
**g__Bilophila**	**7.9649E-8**	2.1107E-6	18279.0	31391.0	22066.0	4788.6	1074.5	4.19
**g__Lachnoclostridium**	**4.0876E-7**	5.3301E-6	28254.0	38501.0	29859.0	19055.0	4483.5	4.25
**g__Blautia**	**4.2081E-7**	5.3301E-6	115230.0	100280.0	200050.0	55958.0	3190.2	5.0
**g__Tuzzerella**	**5.0284E-7**	5.3301E-6	13994.0	12370.0	8608.9	3210.1	848.95	3.84
**g__Muribaculaceae**	**6.1749E-7**	5.3384E-6	67611.0	186700.0	121780.0	347190.0	608370.0	5.47
**g__Ruminococcaceae**	**7.0507E-7**	5.3384E-6	0.0	0.0	0.0	1160.7	1233.6	3.37
**g__GCA_900066575**	**1.5088E-6**	9.9956E-6	7388.5	13444.0	7216.1	2427.5	1021.4	3.83
**g__uncultured**	**2.967E-6**	1.7472E-5	131850.0	178860.0	153170.0	56946.0	60030.0	4.88
**g__Rikenella**	**6.2027E-6**	3.2874E-5	0.0	10201.0	3329.5	3860.1	3826.9	3.87
**Not_Assigned**	**7.7015E-6**	3.7107E-5	15626.0	12661.0	12741.0	2739.2	3860.1	3.92
**g__Butyricicoccus**	**1.5521E-5**	6.8549E-5	2188.7	1896.9	1101.0	391.31	39.795	3.04
**g__Erysipelatoclostridium**	**2.3424E-5**	9.5497E-5	0.0	0.0	0.0	13862.0	4609.5	3.84
**g__Lactobacillus**	**3.6947E-5**	1.3987E-4	955.07	610.18	2235.1	41466.0	23592.0	4.42
**g__Romboutsia**	**6.2724E-5**	2.2162E-4	0.0	1591.8	557.13	126.02	0.0	3.07
**g__Eubacterium_xylanophilum_group**	**8.565E-5**	2.8372E-4	0.0	2692.8	577.02	1585.2	5259.5	3.53
**g__Escherichia_Shigella**	**1.2726E-4**	3.9677E-4	12350.0	14174.0	7163.0	1870.3	179.08	3.85
**g__Mucispirillum**	**1.4253E-4**	4.1967E-4	29700.0	5511.6	19785.0	5717.2	3641.2	4.46
**g__Peptococcus**	**5.2458E-4**	0.0014633	4337.6	1253.5	3840.2	510.7	1492.3	3.33
**g__Colidextribacter**	**6.1405E-4**	0.0016272	38866.0	56800.0	29614.0	13106.0	17788.0	4.36
**g__Family_XIII_UCG_001**	**6.6204E-4**	0.0016709	0.0	0.0	0.0	683.14	1764.2	2.95
**g__Candidatus_Saccharimonas**	**8.1548E-4**	0.0019646	1034.7	1180.6	4510.1	2228.5	2460.6	3.55
**g__Parabacteroides**	**8.8454E-4**	0.0020383	3382.5	2739.2	1910.1	17404.0	902.01	3.92
**g__Alistipes**	**9.7947E-4**	0.002163	65038.0	10725.0	44563.0	24573.0	41221.0	4.55
**g__Faecalibaculum**	**0.0013205**	0.0027994	1870.3	1552.0	10479.0	2666.2	106.12	3.72
**g__Lachnospiraceae_FCS020_group**	**0.001891**	0.0038546	981.6	1545.4	1883.6	391.31	86.222	2.97
**g__Oscillibacter**	**0.0021464**	0.0042133	4191.7	9199.2	7275.8	3959.6	7123.2	3.72
**g__UCG_009**	**0.0022765**	0.0043091	172.44	1246.9	974.97	397.95	1346.4	3.13
**g__Harryflintia**	**0.0030904**	0.0055501	941.81	636.71	53.06	59.692	0.0	2.67
**g__Coriobacteriaceae_UCG_002**	**0.0031416**	0.0055501	915.28	2745.8	20886.0	2639.7	855.59	4.11
**g__UBA1819**	**0.0045984**	0.0076733	0.0	33.162	145.91	676.51	875.48	3.21
**g__Desulfovibrio**	**0.0046329**	0.0076733	3488.7	0.0	1227.0	3820.3	10121.0	4.0
**g__Lachnospiraceae_NK4A136_group**	**0.007086**	0.011352	74575.0	83483.0	48967.0	35643.0	71140.0	4.73
**g__Incertae_Sedis**	**0.0072823**	0.011352	6579.4	7448.2	4801.9	3873.3	2692.8	3.4
**g__Bacteroides**	**0.0082883**	0.012551	250390.0	152170.0	139550.0	247090.0	57828.0	4.98
**g__Eubacterium_nodatum_group**	**0.0087091**	0.012822	344.89	318.36	988.23	1525.5	696.41	2.86
**g__Parasutterella**	**0.016835**	0.024114	0.0	590.29	13928.0	12854.0	7269.2	4.03
**g__Anaerotruncus**	**0.018275**	0.025489	6857.9	11925.0	7103.3	7136.5	2891.7	3.71
**g__NK4A214_group**	**0.018879**	0.025656	0.0	391.31	517.33	1671.4	1804.0	3.05
**g__ASF356**	**0.022563**	0.029718	198.97	0.0	523.96	809.16	809.16	2.62
**g__Clostridia_vadinBB60_group**	**0.023195**	0.029718	570.39	1140.8	2891.7	238.77	537.23	3.42
**g__A2**	**0.02355**	0.029718	411.21	252.03	0.0	1883.6	2473.9	3.28

Supplementary Figure 15

Linear Discriminant Analysis Effect Size (LEfSe)

g-Prevotellaceae UCG001

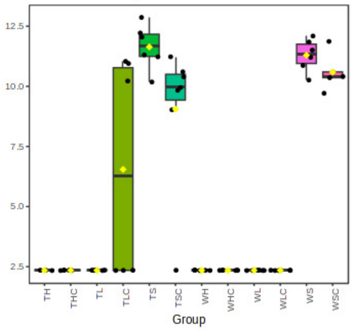

g-Colidextribacter

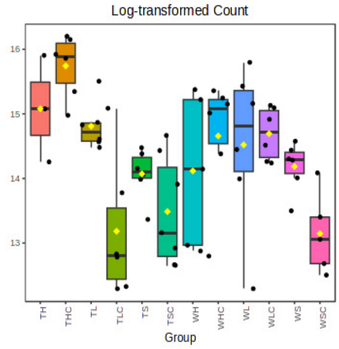

g__Lachnoclostridium

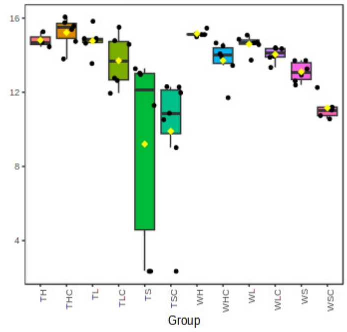

g-Tuzzerella

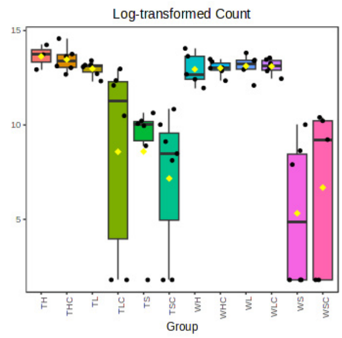

g-GCA 900066575

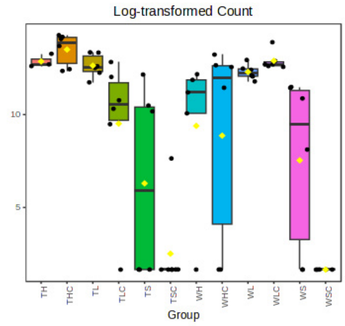

g-Butyricicoccus

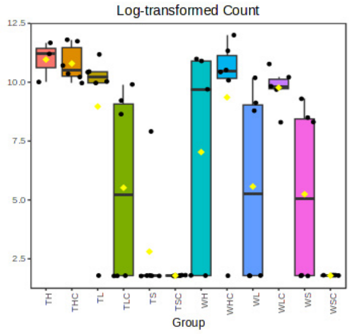

g-Escherichia-Shigella

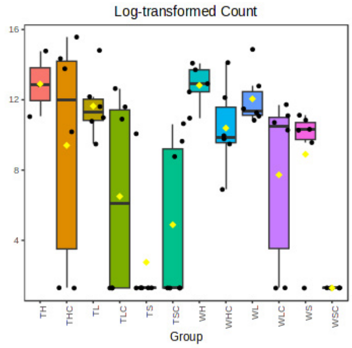

g-Colidextribacter

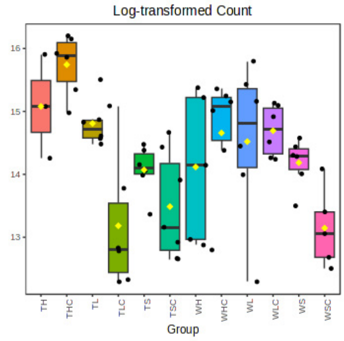

g-Incertae_Sedis

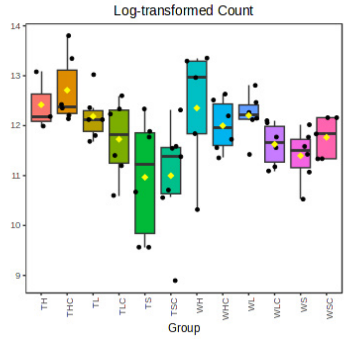

Supplementary Figure 16

SUPPLEMENTARY FIGURES

Supplementary Figure 1

A-The Plots show alpha diversity analysis from WT and TG (APP/PS1) groups, the corresponding Kluskal-Walllis statistical analysis was indicated below.

B-The beta diversity data from WT vs TG , at phylum and Genus level were indicated, using Bray Curtis analysis.

C- The differences WT vs TG were additionally analysed using Linear Discrimination Test (LEfSe), showing only a genus (g-RF39) statistically significantly different in TG

D- The stacked bar plots represent relative abundance profile TG and WT mice groups, at phylum taxonomic rank (top graph); and at genus taxonomic rank (lower graph). In both cases some obvious differences were marked with colour arrows.

E- In addition of the Linear Discrimination Test (LEfSe) presented in Figure 1 we did a Single-factor Statistical Comparison. The data at genus taxonomic rank, confirmed g-RF39 statistically significantly different in TG as indicated the p value.

Supplementary Figure 2

A- The stacked bar plots represent relative abundance profile from sexual mice groups (F, M and MC), at phylum taxonomic rank. Some obvious differences were marked with colour arrows.

B-Plots show beta diversity analysis from sexual mice groups considered (F, M and MC), using Bray Curtis, at phylum level. The corresponding statistical analysis was indicated on the right table, showing p and DFR values.

C-The data from sexual mice groups considered (F, M and MC), were additionally analysed using Linear Discrimination Test (LEfSe), at genus taxonomic rank showing significantly differences in p and FDR values (Note that p values were represented in bold only when with FDR below 0.04.)

Supplementary Figure 3

We additionally analysed F, M and MC sexual groups, using Linear Discrimination Test (LEfSe). At specie taxonomic rank, we found significantly differences in four species as indicated in the table (Note that p values were represented in bold only when with FDR below 0.04.)

-In addition, LDA plots from F, M and MC mice shown more abundant species in each group, when considering LDA >2. The graphics below, represent the log-transformed values from most significantly altered species among groups (*Bacteroides caecimuris* and *Lactobacillis reuteri*).

Supplementary Figure 4

A-Plots show the alpha analysis from the three sexual groups and the two genotypes considered (FW, FT, WS. WSC and TS, TSC), at genus (left) and specie (right) taxonomic rank using Shannon test, using a Kluskal-Wallis. The asterisks (**- $p<0.001$) represent p value when WS and WSC was statistical analysis.

The tables below represent the represent p value and FDR value from Kluskal-Wallis statistical analysis, from each group, in pairs.

B- The stacked bar plots represent the relative abundance profile in FW, FT, WS. WSC and TS, TSC mice groups, at phylum taxonomic rank. Some obvious difference was marked with colour arrow.

Supplementary Figure 5

A-Plots show beta diversity analysis from mice groups feed with each diet, when we additionally considered genotype and sexual groups. Considering genotypes, we represented WS, WH, WL; WSC, WHC, WLC (wild-type); and TS, TH, TL, TSC, THC, TLC (APP/PS1), using Bray Curtis test, at genus taxonomic rank. The corresponding statistical analysis was represented on the right table, representing p and DFR values. (Note that p values were represented in bold only when with FDR below 0.04.)

B. Plots show Linear Discrimination Analysis (LDA) from above mice groups, at genus taxonomic rank, show some genera more abundant in the groups previously considered (Represented only when considering LDA >2).

Supplementary Figure 6

The data from considering groups (WS, WH, WL; WSC, WHC, WLC; and TS, TH, TL, TSC, THC, TLC) were additionally analysed using Linear Discrimination Test (LEfSe). The tables represent the data from phylum and genus taxonomic ranks where p values were represented in bold only when with FDR ≤ 0.04.

Supplementary Figure 7

Additionally, to the Linear Discrimination Test we performed a Single Factor statistical analysis (DESeq2) to reinforce the differences between: male versus female in wild type groups (WS /FW), wild-type versus transgenic (WS/TS), and castrated-males versus males (WS/WSC), all groups in standard food diet (S).

The corresponding statistical analysis were represented in each table, representing p and DFR values. (Note that p values were represented in bold only when with FDR below 0.04.). The graphics below, represent the log-transformed values from most significantly altered genera between groups.

Supplementary Figure 8

A-Plots show the alpha analysis at phylum and genus level, from TG and WT mice groups, using Shannon test, when all diets were considered. The table represent the represent p value and FDR value from Kluskal-Wallis statistical analysis. (Note that p values were represented in bold only when with FDR below 0.03.)

B-C- The stacked bar plots represent relative abundance profile TG and WT mice groups (all diets considered), at phylum taxonomic rank (**B**); and at genus taxonomic rank (**C**). In both cases some obvious differences were marked with colour arrows.

Supplementary Figure 9

A-Plots show the alpha analysis at phylum and genus level, from M and MC mice groups, using Shannon test, when all diets were considered. The table represent the represent p value and FDR value from Kluskal-Wallis statistical analysis. (Note that p values were represented in bold only when with FDR below 0.03.)

B-C- The stacked bar plots represent relative abundance profile M and MC mice groups (all diets considered), at phylum taxonomic rank (**B**); and at genus taxonomic rank (**C**). In both cases some obvious differences were marked with colour arrows.

Supplementary Figure 10

A-Plots show beta diversity analysis from TG and WT mice groups (all diets considered), using Bray Curtis test, at genus taxonomic rank. The corresponding statistical analysis was represented on the right table, representing p and DFR values.

B-Plots show beta diversity analysis from M and MC mice groups, using Shannon test, when all diets were considered, using Bray Curtis test, at genus and featured-level taxonomic rank. The corresponding statistical analysis was represented on the right table, representing p and DFR values. (Note that p values were represented in bold only when with FDR below 0.04.)

C- Plots show Linear Discrimination Analysis (LDA) from M and MC mice groups, at genus taxonomic rank, show some genera more abundant in these groups (Represented only when considering LDA >, or < 2).

The table below represent the additional analysed using Linear Discrimination Test (LEfSe) test, from M and MC. The tables represent the data from phylum and genus taxonomic ranks. The p values were denoted in bold only when with FDR ≤ 0.04.

Supplementary Figure 11

The table represent the additional analysed using Linear Discrimination Test (LEfSe) test, from the mice groups fed with each diet (S, L and H), at genus taxonomic rank. The p values were denoted in bold only when with FDR ≤ 0.04.

Supplementary Figure 12

In addition of the previous Linear Discrimination Test (LEfSe) presented in Supl. Figure 11 we did a Single-factor Statistical Comparison (DEReq2). The data at genus taxonomic rank, was performed between S and H diet, and between L and S diets.

The statistically significant p values were represented in bold only when with FDR ≤ 0.04.

The graphical below represent the log-transformed values from most significantly altered genera when compared with H diet

Supplementary Figure 13

Plots show the alpha analysis at phylum and genus level, from all groups and the different diets considered [WS, WH, WL; WSC, WHC, WLC (wild-type); and TS, TH, TL, TSC, THC, TLC (APP/PS1)], using Shannon test.

The tables represent the represent p value and FDR value from Kluskal-Wallis statistical analysis. (Note that p values were represented in bold only when with FDR below 0.03.)

Supplementary Figure 14

Table show beta diversity analysis from (WS, WH, WL; WSC, WHC, WLC; and TS, TH, TL, TSC, THC, TLC), mice groups, when all diets were considered, using Bray Curtis test, at genus taxonomic rank. The statistical analysis represented in the table, represent p and DFR values. (Note that p values were represented in bold only when with FDR below 0.04.)

Supplementary Figure 15

Table of the Linear Discrimination Test (LEfSe) from (WS, WH, WL; WSC, WHC, WLC; and TS, TH, TL, TSC, THC, TLC), mice groups, at genus taxonomic rank. The statistical analysis represented in the table, represent p and DFR values. (Note that p values were represented in bold only when with FDR below 0.03.)

Supplementary Figure 16

The figures represent the log-transformed values from most significantly altered genera when compared all diets from (WS, WH, WL; WSC, WHC, WLC; and TS, TH, TL, TSC, THC, TLC) mice groups: In this case we represent those in which H diet in transgenic mice (TH or THC) was significantly different from L or S diet in transgenic mice (TS, TSC, TL, TLC).
