# Supplementary material for: Sex hormones and diets rich in polyunsaturated ω-6/ω-3 fatty acids modify microbiota distinctly in a mouse model of Alzheimer’s disease
Source: Gut Microbiome (Camb). 2025 Jun 18;6:e10. doi: 10.1017/gmb.2025.10005 (PMC12284841; doi:10.1017/gmb.2025.10005)
Supplement: Ordoñez-Gutierrez and Wandosell supplementary material [file S2632289725100054sup001.zip › Supplementary Table 1 V2 .pdf]

Supplementary Table 1.

A)- LIPID COMPOSITION

	DIETS		
	**L**	**SF**	**H**
**Total Fat content**	**69** mg/g	**46** mg/g	**73** mg/g

**Omega 6 (ω6)**	mg/100g		
**ω6**	2025	1449	2356
**Omega 3 (ω3)**	mg/100g		
**ω3**	615	119	94

**ω -6/ω -3**	**3.2**	**12.3**	**24.6**

B)- PRIMERS USED:

	Primer characteristics	
**Primer name**	**Complete Sequence**	**Amplified sequence**
**16SV3-V4-CS1**	ACACTGACGACATGGTTCTACACCTACGGGNGGCWGCAG	CCTACGGGNGGCWGCAG
**16SV3-V4-CS2**	TACGGTAGCAGAGACTTGGTCTGACTACHVGGGTATCTAATCC	GACTACHVGGGTATCTAATCC
